# The T-cell leukemia related *rpl10-R98S* mutant traps the 60S export adapter Nmd3 in the ribosomal P site in yeast

**DOI:** 10.1371/journal.pgen.1006894

**Published:** 2017-07-17

**Authors:** Stephanie Patchett, Sharmishtha Musalgaonkar, Andrey G. Malyutin, Arlen W. Johnson

**Affiliations:** 1 Depatment of Molecular Biosciences, the University of Texas at Austin, Austin, Texas, United States of America; 2 Department of Biochemistry and Molecular Biophysics, Columbia University, New York, New York, United States of America; Dana Farber Cancer Institute, UNITED STATES

## Abstract

Mutations in the ribosomal protein Rpl10 (uL16) can be drivers of T-cell acute lymphoblastic leukemia (T-ALL). We previously showed that these T-ALL mutations disrupt late cytoplasmic maturation of the 60S ribosomal subunit, blocking the release of the trans-acting factors Nmd3 and Tif6 in *S*. *cerevisiae*. Consequently, these mutant ribosomes do not efficiently pass the cytoplasmic quality control checkpoint and are blocked from engaging in translation. Here, we characterize suppressing mutations of the T-ALL-related *rpl10-R98S* mutant that bypass this block and show that the molecular defect of *rpl10-R98S* is a failure to release Nmd3 from the P site. Suppressing mutations were identified in Nmd3 and Tif6 that disrupted interactions between Nmd3 and the ribosome, or between Nmd3 and Tif6. Using an *in vitro* system with purified components, we found that Nmd3 inhibited Sdo1-stimulated Efl1 activity on mutant *rpl10-R98S* but not wild-type 60S subunits. Importantly, this inhibition was overcome *in vitro* by mutations in Nmd3 that suppressed *rpl10-R98S in vivo*. These results strongly support a model that Nmd3 must be dislodged from the P site to allow Sdo1 activation of Efl1, and define a failure in the removal of Nmd3 as the molecular defect of the T-ALL-associated *rpl10-R98S* mutation.

## Author summary

The ribosome is a large and structurally complex macromolecular machine, responsible for synthesizing proteins in all living cells, across all domains of life. The correct assembly of ribosomes is important for their ability to faithfully decode messenger RNAs and synthesize proteins. The insertion of the ribosomal protein Rpl10 into the ribosome completes the catalytic center of the large subunit and is necessary for the removal of the assembly factors Nmd3 and Tif6, which allows the subunit to participate in translation. The insertion of Rpl10 is monitored by proteins that mimic translation factors during a quality control check for ribosome function. Ribosomes containing mutations in Rpl10 associated with pediatric T-cell leukemia fail in this quality control check and prevent the removal of Tif6 and Nmd3. However, it was not known how these mutations in Rpl10 block the quality control check. We recently presented the structure of Nmd3 and Tif6 on the large ribosomal subunit from yeast. In this work, we take advantage of our recent structural work and use a combination of genetic and biochemical techniques to delineate the molecular defect in the ribosome when Rpl10 is mutated.

## Introduction

The eukaryotic ribosome is composed of 4 ribosomal RNA molecules and roughly 80 ribosomal proteins that come together to form the large (60S) and small (40S) subunits that make up mature 80S ribosomes. Eukaryotic ribosome biogenesis is an intricate assembly pathway that requires the assistance of hundreds of trans-acting factors [1–6], and although ribosomes catalyze protein synthesis in the cytoplasm, this process takes place predominantly in the nucleus. Each nascent subunit must therefore be exported through the nuclear pore complex, a process that is dependent on the export receptor Crm1 [7–9]. In 60S export, Crm1 is recruited via the export adapter Nmd3, which contains a leucine-rich nuclear export signal (NES) [10–12]. In yeast, additional factors assist in 60S export, including the non-canonical export receptor Arx1 [13,14], the mRNA export receptor Mex67-Mtr2 [15], Ecm1 [16], and Bud20 [17]. However, only Nmd3 appears to be universally conserved in eukaryotes as a dedicated 60S export factor [9–12]. Nascent 60S subunits entering the cytoplasm are bound by additional trans-acting factors, including the anti-association factor Tif6 (eIF6 in mammalian cells) [18]. Trans-acting factors bound to subunits in the cytoplasm block important functional regions and are thought to prevent immature subunits from engaging prematurely with the translational machinery (reviewed in [19]). For example, Tif6 binds to the inter-subunit face of the 60S subunit and sterically block 40S joining [20,21]. In addition, our lab and others have recently shown that Nmd3 occupies the A, P, and E sites of the tRNA channel where it would prevent association of ligands for these sites [22,23]. Newly exported subunits also lack several critical ribosomal proteins, including Rpl10 [6,24]. [Note that Rpl10 is also named uL16 in the new nomenclature [25], however, we use Rpl10 here for clarity with genetic mutations]. As such, pre-60S particles enter the cytoplasm in an inactive state and must undergo cytoplasmic maturation, a process of shedding trans-acting factors and assembling the final ribosomal proteins [26,27].

**Fig 1:**
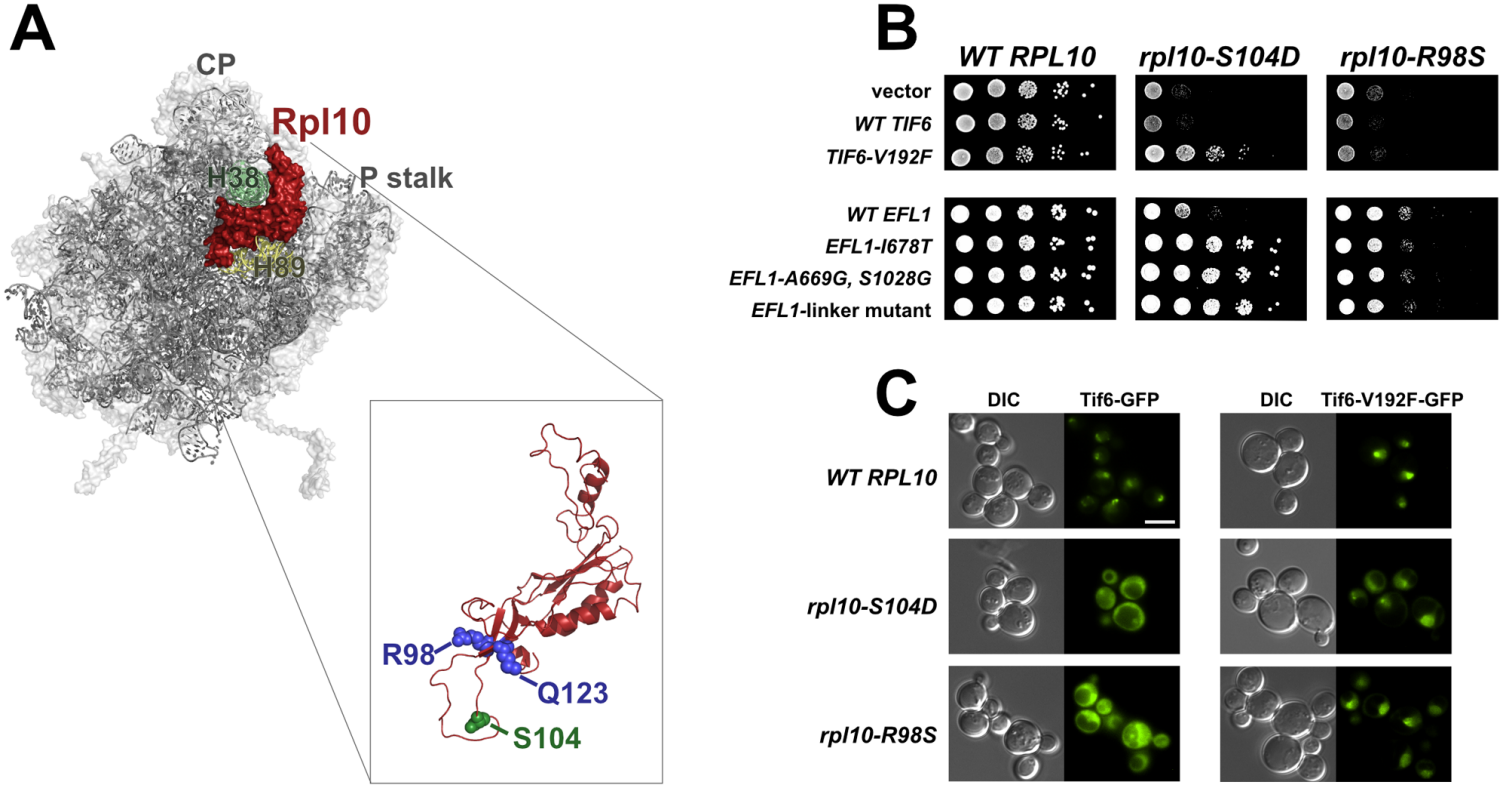
The *rpl10-R98S* defect is not suppressed by Tif6 release. **A)** The position of Rpl10 in the crown view of the 60S subunit. The central protuberance (CP), helix 38 (H38) and helix 89 (H89) are indicated. A cartoon of Rpl10 structure showing amino acids mutated in T-ALL (blue) (From PDB files 5ANB, 3U5D and 3U5E). **B)** 10-fold serial dilutions of the glucose repressible *RPL10* strain AJY3373 (P_GAL_*-RPL10*) harboring *WT RPL10*, *rpl10-S104D*, or *rpl10-R98S* vector, and either empty vector or the indicated *TIF6* or *EFL1* plasmids. Cells were spotted onto glucose-containing selective media to repress genomic *RPL10*. **C)** Tif6-GFP and Tif6-V192F-GFP localization monitored by fluorescence microscopy in *WT RPL10*, *rpl10-S104D*, and *rpl10-R98S* cells. AJY2766 (P_GAL_*-RPL10*, *TIF6-GFP*) and AJY3941 (P_GAL_*-RPL10*, *TIF6-V192F-GFP*) expressing WT *RPL10*, *rpl10-S104D*, or *rpl10-R98S* from plasmids were grown in the presence of glucose to repress genomic *RPL10*. DIC, differential interference contrast. Scale bar, 5μm.

60S cytoplasmic maturation events take place in a highly ordered pathway [28], and the final two known steps include the release of Tif6 by Efl1 and Sdo1 [18,29,30] and the release of Nmd3, dependent on the GTPase Lsg1 [24,31]. After these steps, 60S subunits are competent to participate in translation. Genetic analyses place the release of Nmd3 downstream of and dependent on the prior release of Tif6 [28,32,33]. However, recent structures of pre-60S particles call that order of events into question [34]. A critical event that takes place upstream of Tif6 and Nmd3 release in cytoplasmic maturation is the addition of ribosomal protein Rpl10 [24,35], which binds in a cleft between the central protuberance and the P stalk of the 60S subunit (Fig 1A). An internal loop of Rpl10 (referred to here as the P site loop) extends toward the catalytic center of the ribosome where it completes the peptidyl-transferase center and makes contact with the acceptor stem of P site ligands, including tRNA, during translation [36]. Extensive genetic analyses have demonstrated the importance of Rpl10 in 60S cytoplasmic maturation. Specifically, the P site loop of Rpl10 is required for the release of both Tif6 and Nmd3 [32,37].

While the mechanism of Nmd3 release is not understood, Tif6 release has been shown to depend on Sdo1 binding in the ribosomal P site where it cooperates with the GTPase Efl1, a paralog of the translation elongation factor EF2, to evict Tif6 [34]. Although Sdo1 has been shown to stimulate Efl1 GTPase activity on 60S subunits [33], structural studies suggest that Efl1 may release Tif6 by competing with it for binding, independent of GTPase activity [34]. We and others have proposed that the release of Tif6 by Efl1 and Sdo1 acts as a quasi-functional “test drive” of nascent 60S subunits in which translation factor-mimics assess the functionality of newly made subunits. Subunits that fail the test drive (including Rpl10 mutants) retain Tif6 and Nmd3, and are prevented from engaging in translation [32,34].

Deficiencies in the human Sdo1 protein, the Shwachman-Bodian-Diamond syndrome protein (SBDS), are associated with Shwachman-Diamond syndrome (SDS), a congenital disorder characterized by bone marrow failure and exocrine pancreatic insufficiency [38]. Interestingly, SDS is also associated with an elevated risk of myelodysplastic syndrome and acute myeloid leukemia development [39]. In addition, recurring mutations in *RPL10* are associated with pediatric T-cell acute lymphoblastic leukemia (T-ALL) in humans, with the most commonly identified mutation being *rpl10-R98S* [40]. These mutations in T-ALL, like those in SDS, block the release of Tif6 and Nmd3. Together, these findings suggest that defects in late 60S cytoplasmic maturation may contribute to carcinogenesis.

Mutations in *RPL10* are thought to block late 60S maturation by specifically impairing the Efl1- and Sdo1-dependent release of Tif6. A previous mutational analysis of the P site loop of Rpl10 in yeast identified a class of mutants, typified by *rpl10-S104D*, that arrest ribosome biogenesis by failing to promote the Efl1-dependent release of Tif6 [32]. This arrest appears similar to that observed in *sdo1* mutants as wells as in T-ALL-associated *rpl10-R98S* mutants. The *RPL10* mutations also appear similar to disease-related mutations in *SBDS* in their impact on the binding affinity of Sdo1 to 60S subunits *in vivo* [34] and to mature ribosomes *in vitro* [41,42]. While reduced binding of Sdo1 to 60S subunits could explain the defect in Tif6 release, an understanding of how this leads to a block in Nmd3 release is unknown. Moreover, how Tif6 release is coordinated with Nmd3 release is not well understood.

In this work, we explore the molecular defect of the T-ALL related *rpl10-R98S* mutant in ribosome assembly using *Saccharomyces cerevisiae*. Despite phenotypic similarity, the *rpl10-R98S* mutant blocks 60S biogenesis by a mechanism that is distinct from the P loop *rpl10-S104D* mutant. We show that the molecular defect of the T-ALL associated *rpl10-R98S* mutation is a failure to release Nmd3 from the P site, thereby preventing the release of both Nmd3 and Tif6.

## Results

### The T-ALL related *rpl10-R98S* mutation blocks 60S biogenesis by a mechanism distinct from other *rpl10* mutations

Mutations in *RPL10* were identified as driver-mutations in pediatric T-cell acute lymphoblastic leukemia (T-ALL) [40]. These mutations occurred almost exclusively at residue arginine 98 (R98), with the exception of one patient harboring the Q123P mutation, which lies adjacent to R98 at the base of the P site loop of the Rpl10 (Fig 1A). We previously showed that these T-ALL mutants exhibited a large subunit biogenesis defect in which Tif6 and Nmd3 release were blocked [40]. This phenotype appeared similar to the defect observed in a class of Rpl10 P site loop mutants, typified by the *rpl10-S104D* mutant, identified in an earlier mutational analysis of *RPL10* [32]. We therefore anticipated that the molecular defect of the T-ALL related *rpl10-R98S* mutation was the same as that caused by *rpl10-S104D* and would be suppressed by the same mutations. We previously showed that *rpl10-S104D* can be suppressed by the dominant *TIF6* mutant, *TIF6-V192F*, which belongs to a class of mutations that map to the 60S-binding surface of Tif6 [32]. Such dominant mutations in *TIF6* also suppress the growth defects due to loss of Efl1 [18,29] or Sdo1 [30] by weakening Tif6 affinity for the 60S subunit, thereby uncoupling the release of Tif6 from the requirement for Efl1 and Sdo1. We also identified mutations in *EFL1* itself that suppressed *rpl10-S104D*. These mutations are thought to predispose Efl1 to undergo a conformational change that normally requires proper signaling through Sdo1 binding in the P site [32]. Thus, a probable explanation for the effect of *rpl10-S104D* on the release of Tif6 is that the Rpl10 mutant ribosome is defective for Sdo1 binding. Indeed, we recently found that both *rpl10-S104D* and *rpl10-R98S* ribosomes had a weakened affinity for Sdo1 compared to wild-type ribosomes *in vitro* [41,42], while others have also demonstrated that the T-ALL associated *RPL10* alleles impair Sdo1 binding to the 60S subunit *in vivo* [34].

Unexpectedly, we found that although *TIF6-V192F* and *EFL1* mutants could suppress the *rpl10-S104D* growth defect, they could not suppress the *rpl10-R98S* defect (Fig 1B). This result was surprising because both *rpl10* mutants are defective for Tif6 release, and the lower affinity of Tif6-V192F for 60S should promote its release. We considered two possibilities to explain the difference between the two *rpl10* mutations: 1) Tif6-V192F remains trapped on *rpl10-R98S* pre-60S subunits or 2) Tif6-V192F is released from *rpl10-R98S* subunits, but its release does not suppress the full biogenesis defect. To determine whether Tif6-V192F was released, we monitored the localization of Tif6-GFP versus Tif6-V192F-GFP in *rpl10-R98S* cells, and in *rpl10-S104D* cells as a control. The nuclear localization of Tif6 in wild-type cells reflects its ability to be released from pre-60S subunits in the cytoplasm and subsequently recycled to the nucleus [18,28,30,43]. As previously reported [32,40], wild-type Tif6-GFP was mislocalized to the cytoplasm in both *rpl10* mutants, indicating a failure in Tif6 release from the 60S subunit (Fig 1C, left panels). Also in agreement with previous observations, we found that the steady state localization of Tif6-V192F-GFP was nuclear in *rpl10-S104D* cells, demonstrating that it recycles back to the nucleus [32]. Unexpectedly, we found that Tif6-V192F-GFP was also localized to the nucleus in *rpl10-R98S* cells (Fig 1C, row 3). These results indicate that *TIF6-V192F* can bypass the block in its release in both *rpl10* mutants. However, while its release could suppress *rpl10-S104D*, the release of Tif6-V192F was not sufficient to suppress the *rpl10-R98S* growth defect.

### The *rpl10-R98S* mutant is suppressed by mutations in *NMD3* and in *TIF6*

**Fig 2:**
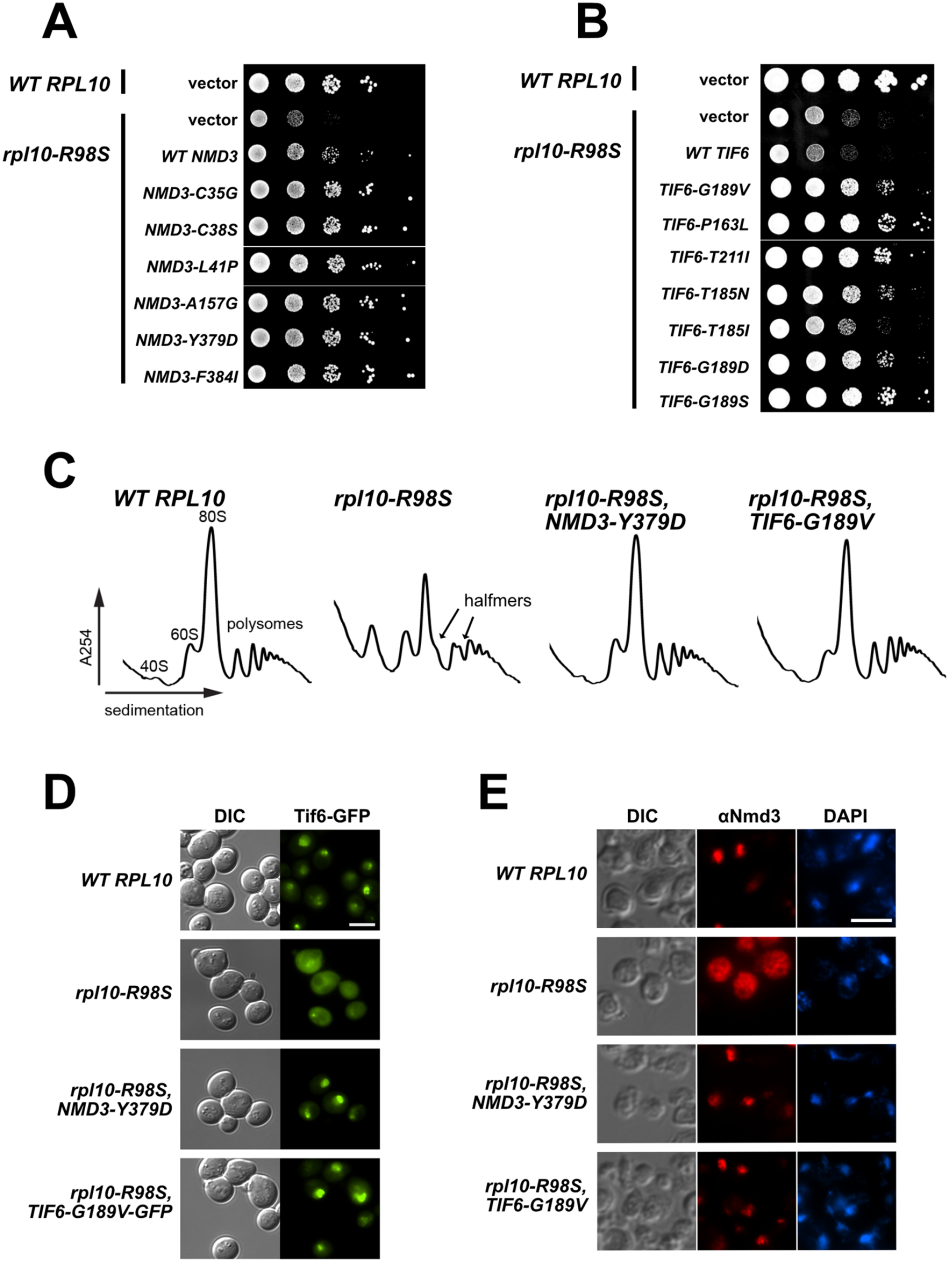
The *rpl10-R98S* defect is suppressed by mutations in *NMD3* and in *TIF6*. **(A, B)** 10-fold serial dilutions of strain AJY3373 (P_GAL_*-RPL10*) expressing either WT *RPL10* or *rpl10-R98S* and the indicated alleles of *NMD3* (A) or *TIF6* (B), all from centromeric vectors, plated onto glucose-containing selective media. **C)** Polysome profile analysis of *WT RPL10* (AJY2781), *rpl10-R98S* (AJY2784), and *rpl10-R98S* cells containing glucose-repressible *RPL10* (pDEGQ2) and either the *NMD3-Y379D* (AJY2846) or *TIF6-G189V* (AJY2848) suppressing allele in the genome. Extracts were prepared after repression of wild-type *RPL10* by growth in glucose-containing media. **D)** Tif6 localization monitored by fluorescence microscopy in cells containing genomic *TIF6-GFP* and either *WT RPL10* (AJY3937) or *rpl10-R98S* (AJY3938) as the sole copy. Suppressed *rpl10-R98S* cells contained either *NMD3-Y379D* (AJY3939) or *TIF6-G189V-GFP* (AJY3940) in the genome. Scale bar, 5μm. **E)** Nmd3 localization monitored by indirect immunofluorescence, following treatment with LMB, in P_GAL_*-RPL10* cells containing an LMB-sensitive crm1 allele (AJY1958) and either wild-type *RPL10* or *rpl10-R98S* plasmid. Suppressed *rpl10-R98S* cells contained either *NMD3-Y379D* (AJY3909) or *TIF6-G189V (AJY3943)* in the genome. Scale bar, 5μm.

The results described above suggest that while the *rpl10-S104D* mutant is specifically defective in the Efl1-dependent release of Tif6, the *rpl10-R98S* defect is more complex. To gain insight into the mechanism by which the *rpl10-R98S* mutant stalls 60S biogenesis, we identified extragenic suppressors of the *rpl10-R98S* growth defect. We isolated multiple independently-derived spontaneous suppressors of *rpl10-R98S* that appeared readily as fast-growing colonies among the severely growth-impaired *rpl10-R98S* background. Because Nmd3 release, in addition to Tif6 release, is impaired in *rpl10-R98S* cells, we considered the possibility that mutations in Nmd3 that promote its release might suppress the *rpl10-R98S* growth defect. The idea that suppressing mutations could be identified in *NMD3* was also supported by our earlier observation that ectopic expression of *WT NMD3* partially suppressed the *rpl10-R98S* growth defect [40] (Fig 2A). Thus, we began by sequencing the *NMD3* genomic locus in each of the suppressors. Remarkably, of 18 spontaneous suppressors analyzed, 16 contained mutations in *NMD3* (Table 1) (the *NMD3-Y379D* mutant was described previously [42]). To confirm that the *NMD3* mutations were responsible for *rpl10-R98S* suppression, we expressed the mutants ectopically and found that all *NMD3* alleles tested were dominant suppressors of *rpl10-R98S* (Fig 2A). Because the remaining two spontaneous suppressors contained wild-type *NMD3*, we performed whole genome sequencing on one isolate to identify mutations elsewhere in the genome that could be responsible for *rpl10-R98S* suppression. SNP analysis revealed a mutation in *TIF6*, resulting in a Gly to Val change at amino acid position 189 (*TIF6-G189V*). Sanger sequencing of the *TIF6* locus from the final remaining suppressor identified the *TIF6-T185A* allele. To identify additional mutations in *TIF6* that could suppress *rpl10-R98S*, we performed random PCR mutagenesis of *TIF6* and identified additional suppressing mutations at amino acid positions T185 and G189 as well as mutations at P163 and T211 (Fig 2B and Table 2). Thus, despite the fact that we had initially discounted *TIF6* as a target of suppressing mutations because *TIF6-V192F* did not suppress *rpl10-R98S*, other alleles of *TIF6* are suppressors. Unlike the case for *NMD3*, ectopic expression of WT *TIF6* did not suppress *rpl10-R98S* and, in fact, slightly inhibited growth (Fig 2B). On the other hand, Tif6-V192F, which has weakened affinity for 60S subunits, neither inhibited nor suppressed the growth of *rpl10-R98S* cells, suggesting that the mechanism by which the *TIF6* suppressing mutations acted was not through weakening the interaction between Tif6 and the ribosome.

**Table 1:** *NMD3* alleles identified as spontaneous suppressors of *rpl10-R98S*.

*NMD3* allele		
a.a. change	nt change	domain
C35G	T103G	N-terminal
C38S	T112A	N-terminal
L41P	T122C	N-terminal
D44E	C132A	N-terminal
R88P	G263C	N-terminal
A157G	C470G	eL22-like
D198G	A593G	eL22-like
N205Y	A613T	eL22-like
K224E	A670G	eL22-like
S245F	C734T	eL22-like
W302C	G906C	eIF5A-like
S377L	C1130T	eIF5A-like
Y379D	T1135G	eIF5A-like
F384I	T1150A	eIF5A-like
Y391D	T1171G	eIF5A-like

**Table 2:** *TIF6* alleles identified as suppressors of *rpl10-R98S*.

*TIF6* allele		
a.a. change	nt change	Number of isolates
P163L	C488T	1
T185A	A553G	6
T185I	C554T	1
T185N	C554A	1
G189V	G566T	1
G189D	G566A	1
G189S	G565A	2
T211I	C632T	1

### *rpl10-R98S* suppressors restore Tif6 and Nmd3 release

Suppression of the *rpl10-R98S* growth defect implies that the *NMD3* and *TIF6* alleles identified above restore ribosome biogenesis by allowing the release of Tif6 and Nmd3 from mutant 60S subunits. We examined ribosomal subunit levels from *rpl10-R98S* cells harboring representative *NMD3* and *TIF6* mutants using sucrose density gradient sedimentation. This analysis confirmed that the biogenesis defect of *rpl10-R98S* cells was suppressed by either *NMD3-Y379D* or *TIF6-G189V*, evidenced by the apparent wild-type 60S:40S ratio and the disappearance of halfmer polysomes (Fig 2C). We also checked the localization of both Tif6 and Nmd3 in *rpl10-R98S* mutant cells suppressed with either *NMD3-Y379D* or *TIF6-G189V*. To monitor Tif6 localization, a C-terminal GFP tag was integrated into the *TIF6* genomic locus in wild-type *RPL10* cells, *rpl10-R98S* cells, and *rpl10-R98S* cells harboring the suppressing *NMD3-Y379D* or *TIF6-G189V* alleles in the genome. Again, we saw that Tif6 was mislocalized to the cytoplasm in *rpl10-R98S* cells (Fig 2D, compare row 2 to row 1), consistent with a failure in its release from pre-60S subunits. However, the localization of Tif6-GFP was restored in *rpl10-R98S* cells containing *NMD3-Y379D* (Fig 2D, row 3). In addition, although wild-type Tif6-GFP was mislocalized to the cytoplasm in *rpl10-R98S* cells, Tif6-G189V-GFP localization was restored to the nucleus (Fig 2D, row 4). Because Nmd3 is distributed throughout the cytoplasm during steady-state conditions [44], we monitored the localization of Nmd3 in strains containing the leptomycin B (LMB)-sensitive *crm1-T539C* mutation [45]. Treatment with LMB in this mutant background blocks Crm1-dependent nuclear export, and the subsequent nuclear accumulation of Nmd3 in wild-type *RPL10* cells reflects its ability to be released from pre-60S subunits in the cytoplasm and shuttle back to the nucleus [10,11] (Fig 2E, row 1). As previously reported, Nmd3 could shuttle to the nucleus in wild-type *RPL10* cells, but was distributed throughout the cytoplasm in *rpl10-R98S* cells (Fig 2E, row 2) [40]. However, Nmd3-Y379D could shuttle to the nucleus in *rpl10-R98S* cells, and wild-type Nmd3 shuttling was restored in *rpl10-R98S* cells expressing Tif6-G189V (Fig 2E, rows 3–4). These results demonstrate that the *NMD3-Y379D* and *TIF6-G189V* suppressors simultaneously restore the localization of both Tif6 and Nmd3. The ability of mutant Nmd3 to restore the release and nuclear shuttling of Tif6 was a surprising result because previous genetic analyses suggested that the release of Nmd3 was downstream of the release of Tif6 [28,32]. The results shown here are the first indication that a block in Nmd3 release could inhibit Tif6 release.

### Two classes of *TIF6* mutations affect Tif6 release by distinct mechanisms

**Fig 3:**
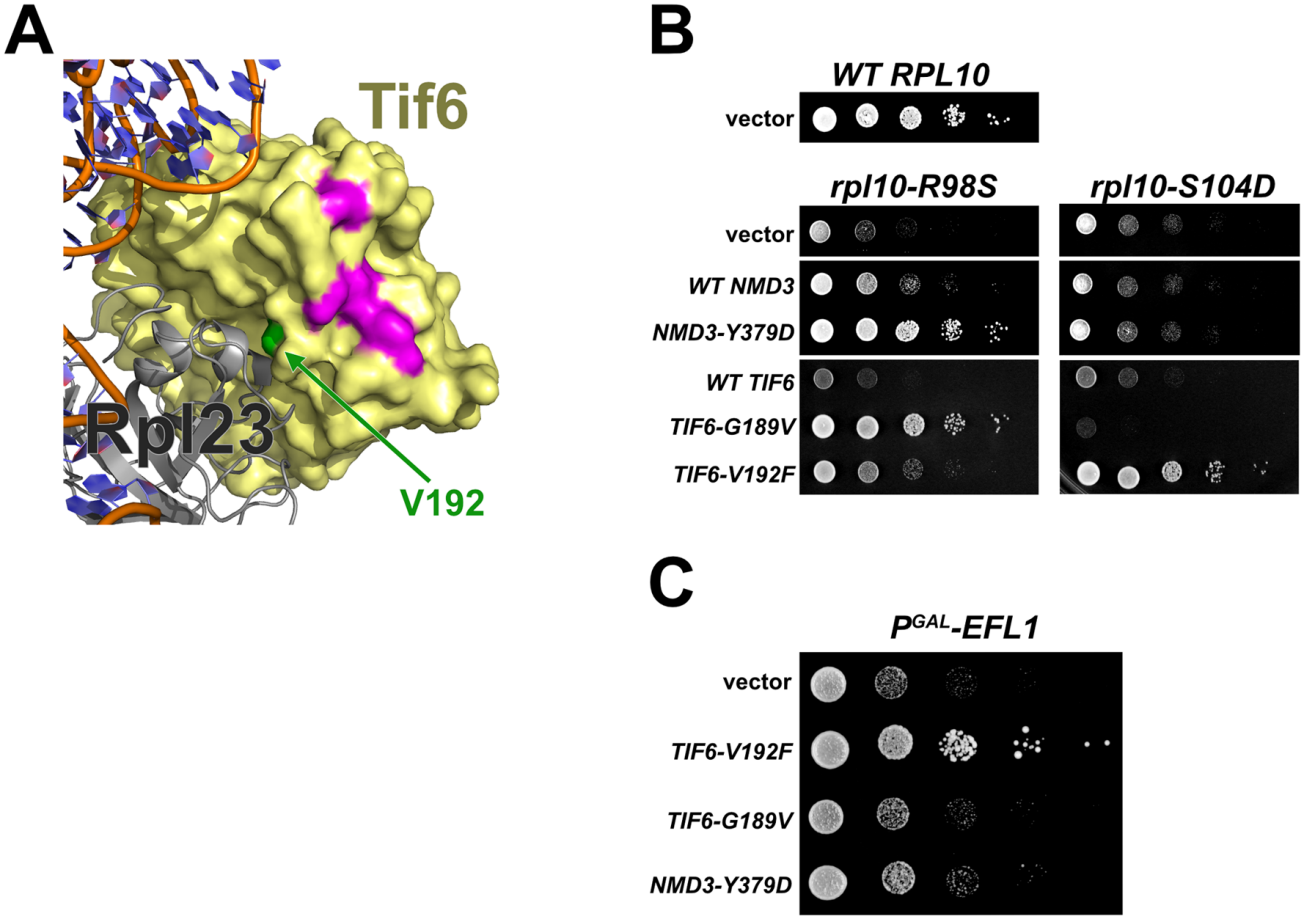
Allele specificity between *RPL10* and *TIF6*. **A)** Structure showing Tif6 bound to Rpl23 on the 60S subunit. Tif6 residue V192 (green) is located at the interface between Tif6 and Rpl23, while Tif6 residues mutated in *rpl10-R98S* suppressors (magenta) are clustered in a nearby region distinct from the interface with the 60S subunit. (Assembled from PBD file 5ANB) **B)** 10-fold serial dilutions of AJY3373 (P_GAL_*-RPL10*) containing *WT RPL10*, *rpl10-R98S*, or *rpl10-S104D* vectors, and either empty vector or the indicated alleles of *NMD3* on centromeric vectors or *TIF6* alleles on high copy vectors. Cells were spotted onto glucose-containing selective media to repress genomic *RPL10*. **C)** Serial dilutions of the glucose repressible *EFL1* strain AJY2981 (P_GAL_*-EFL1*) containing empty vector or the indicated *TIF6* or *NMD3* plasmids. Cells were spotted onto glucose-containing selective media to repress Efl1.

Tif6 binds to the 60S subunit primarily through Rpl23/uL14 [21], and mutations such as Tif6-V192F, map to this interface and weaken the binding of Tif6 to the subunit [18,29,30]. Unlike Tif6-V192F, the four residues in Tif6 that are mutated in the *rpl10-R98S* suppressors cluster together on a surface of Tif6 that is close to, but distinct from, the Tif6-60S interface (Fig 3A). The position of the *rpl10-R98S* suppressing mutations in the Tif6 structure suggests that they act by a different mechanism. Intriguingly, the *rpl10-R98S* suppressing mutations in *TIF6* cluster in a region that interacts directly with Nmd3 [22] (see below).

Because this distinct class of *TIF6* alleles (and *NMD3* alleles) could restore Tif6 release in *rpl10-R98S* cells, we wondered whether their mechanism(s) of suppression was specific to *rpl10-R98S*, or if they could suppress other mutants that block Tif6 release. As discussed earlier, *rpl10-S104D* mutants are also defective for Tif6 release. We transformed *rpl10-S104D* cells with either WT or mutant *TIF6* or *NMD3* vectors. While *NMD3-Y379D* and *TIF6-G189V* dramatically improved *rpl10-R98S* cell growth, neither mutant could suppress the *rpl10-S104D* growth defect (Fig 3B). Depletion of Efl1 also prevents Tif6 release [18,29]. We transformed a glucose-repressible *EFL1* strain (P_GAL_-*EFL1*) with either WT or mutant *TIF6* or *NMD3* vectors and plated onto glucose-containing medium. Again, we found that while *TIF6-V192F* could suppress Efl1 depletion, neither *NMD3-Y379D* nor *TIF6-G189V* suppressed the loss of Efl1 (Fig 3C). These results suggest that the mechanism(s) by which *NMD3-Y379D* and *TIF6-G189V* restored Tif6 release in *rpl10-R98S* cells is not a general Tif6-promoting mechanism, but specifically bypasses the *rpl10-R98S* defect.

### Suppressors of *rpl10-R98S* alter the Nmd3-60S interaction

**Fig 4:**
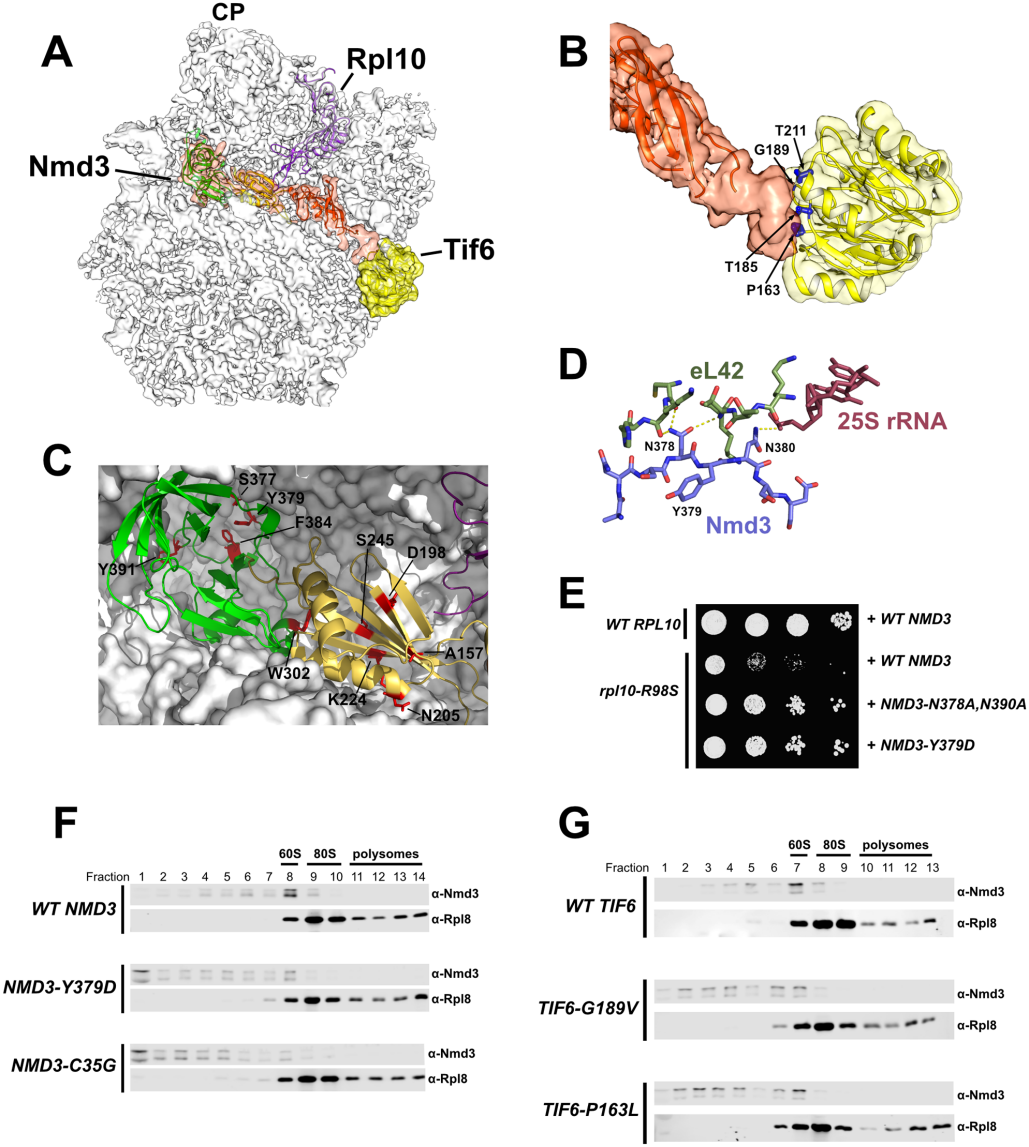
*rpl10-R98S* suppressors alter the interaction between Nmd3 and 60S. **A)** Structure of the yeast 60S subunit in complex with Nmd3 (orange density) and Tif6 (yellow density). The eIF5A-like domain of Nmd3 (green ribbon) occupies the E site and associates with the L1 stalk, while the eL22-like domain (yellow ribbon) occupies the P site and comes into contact with the P site loop of Rpl10 (purple). The N-terminal domain of Nmd3 (orange) contacts Tif6. (From PDB 5T62) **B)** Zoomed view of Tif6 and Nmd3 N-terminal domain. The Tif6 residues mutated in *rpl10-R98S* suppressors (blue) cluster at the Tif6-Nmd3 interface. **C)** Zoomed view of Nmd3 eIF5A- and eL22-like domains. Nmd3 residues mutated in *rpl10-R98S* suppressors (red) map to the Nmd3-60S interface. **D)** Detail of a portion of the Nmd3-60S interface showing hydrogen bonds between Nmd3 residues N378 and N390 with eL42 and 25S rRNA, respectively. **E)** 10-fold serial dilutions of AJY3373 (P_GAL_*-RPL10*) expressing *WT RPL10* or *rpl10-R98S*, and either WT *NMD3* or the indicated *NMD3* mutant from vectors, as indicated. Cells were spotted onto glucose-containing selective media. **F-G)** Sucrose gradient sedimentation of Nmd3. Extracts were fractionated by sucrose gradient sedimentation and the position of Nmd3 in gradients was monitored by Western blotting using anti-Nmd3 antibody. Anti-Rpl8 was used to monitor the position of 60S subunits. **F)** Extracts were prepared from AJY3249 (P_GAL_*-NMD3*) cells expressing WT (pAJ409) or mutant *NMD3* (pAJ2805 or pAJ3609) plasmid as the sole copy. **G)** Extracts were prepared from AJY1700 (tif6Δ) cells expressing either wild-type (pAJ2846) or mutant *TIF6* (pAJ2833 or pAJ3401) as the sole copy.

We recently resolved the structure of Nmd3 on the 60S subunit [22], providing important insights into how Rpl10, Nmd3, and Tif6 interact. Nmd3 consists of three domains: an eIF5A-like domain associates with the L1 stalk and occupies the ribosomal E site, an eL22-like domain occupies the P site and displaces the Rpl10 P site loop from its observed position in mature ribosomes (Fig 4A), while the N-terminal domain extends from the P site toward the SRL and contacts Tif6, providing a direct link between Nmd3 and Tif6 (Fig 4A and 4B). That we could resolve the N-terminal domain of Nmd3 only in the presence of Tif6 suggests that Tif6 stabilizes Nmd3 through their direct interaction. Importantly, all *TIF6* alleles that suppress *rpl10-R98S* can be mapped to this Tif6-Nmd3 interface (Fig 4B). In addition, several of the *rpl10-R98S* suppressing mutations in *NMD3* also occur in the zinc-binding N-terminal domain that contacts Tif6 (Table 1). Although the resolution of the N-terminal 39 residues of Nmd3 was not sufficient to unambiguously trace chains, it is likely that the mutated residues within this domain of Nmd3 affect its interaction with Tif6.

In addition to mutations in the N-terminal domain of Nmd3, the majority of mutations that suppressed *rpl10-R98S* occurred in the eL22- and eIF5A-like domains of Nmd3, and mapped to the Nmd3-60S interface (Table 1, Fig 4C). We therefore considered the possibility that these mutations in Nmd3 might alter its interaction with the 60S subunit. Although residue Y379, (mutated in the *NMD3-Y379D* suppressor) does not make a direct interaction with the ribosome, it is located between N378 and N380, which make hydrogen bonds to eL42 and 25S rRNA, respectively (Fig 4D). The substitution of aspartate for tyrosine at position 379 likely destabilizes these interactions, leading to suppression of *rpl10-R98S*. To test this idea, we made alanine substitutions in N378 and N380 to directly disrupt their interactions with the ribosome and asked whether this mutant, *NMD3-N378A*, *N390A*, could suppress the *rpl10-R98S* growth defect. Indeed, the *NMD3-N378A*, *N390A* mutant suppressed the *rpl10-R98S* growth defect to a similar degree as *NMD3-Y379D* (Fig 4E).

To further test if the Nmd3 mutants identified as *rpl10-R98S* suppressors altered the interaction between Nmd3 and the 60S subunit, we compared the co-sedimentation of wild-type and mutant Nmd3 with 60S subunits. We tested the eIF5A-like domain mutant, *NMD3-Y379D*, and an N-terminal domain mutant, *NMD3-C35G*, both of which could complement in the absence of wild-type *NMD3* (S1 Fig). We found that while wild-type Nmd3 sedimented predominantly in the 60S-containing fractions and was absent from the top of the gradient (indicating that very little free protein was present in cells), both Nmd3-Y379D and Nmd3-C35G mutant proteins were enriched in the free protein fractions at the top of the gradients (Fig 4F). A larger population of free protein relative to 60S-bound Nmd3 could result from a weakened affinity of Nmd3 for the 60S subunit, or from an increased rate of Nmd3 release. While sedimentation cannot distinguish between these possibilities, each scenario suggests an altered interaction between Nmd3 and the 60S subunit.

Nmd3-C35G is predicted to disrupt the stabilizing interaction between Nmd3 and Tif6. To further test the significance of this interaction, we asked if the *rpl10-R98S* suppressing *TIF6* mutations had an impact on Nmd3 sedimentation. We compared Nmd3 sedimentation in cells expressing either wild-type or mutant *TIF6* as the sole copy. We found that both mutants tested (*TIF6-G189V* and *TIF6-P163L*) could complement in the absence of wild-type *TIF6* (S2 Fig). However, while the majority of Nmd3 was found in the 60S fraction in wild-type *TIF6* cells, Nmd3 was distributed in 60S fractions as well as lighter fractions in *TIF6-G189V* and *TIF6-P163L* cells (Fig 4G). This altered Nmd3 sedimentation observed in *TIF6* mutants suggests that, like *rpl10-R98S* suppressing mutations in *NMD3*, mutations in *TIF6* alter the interaction of Nmd3 with the 60S subunit.

### Nmd3 inhibits Efl1 activation on *rpl10-R98S* 60S subunits *in vitro*

**Fig 5:**
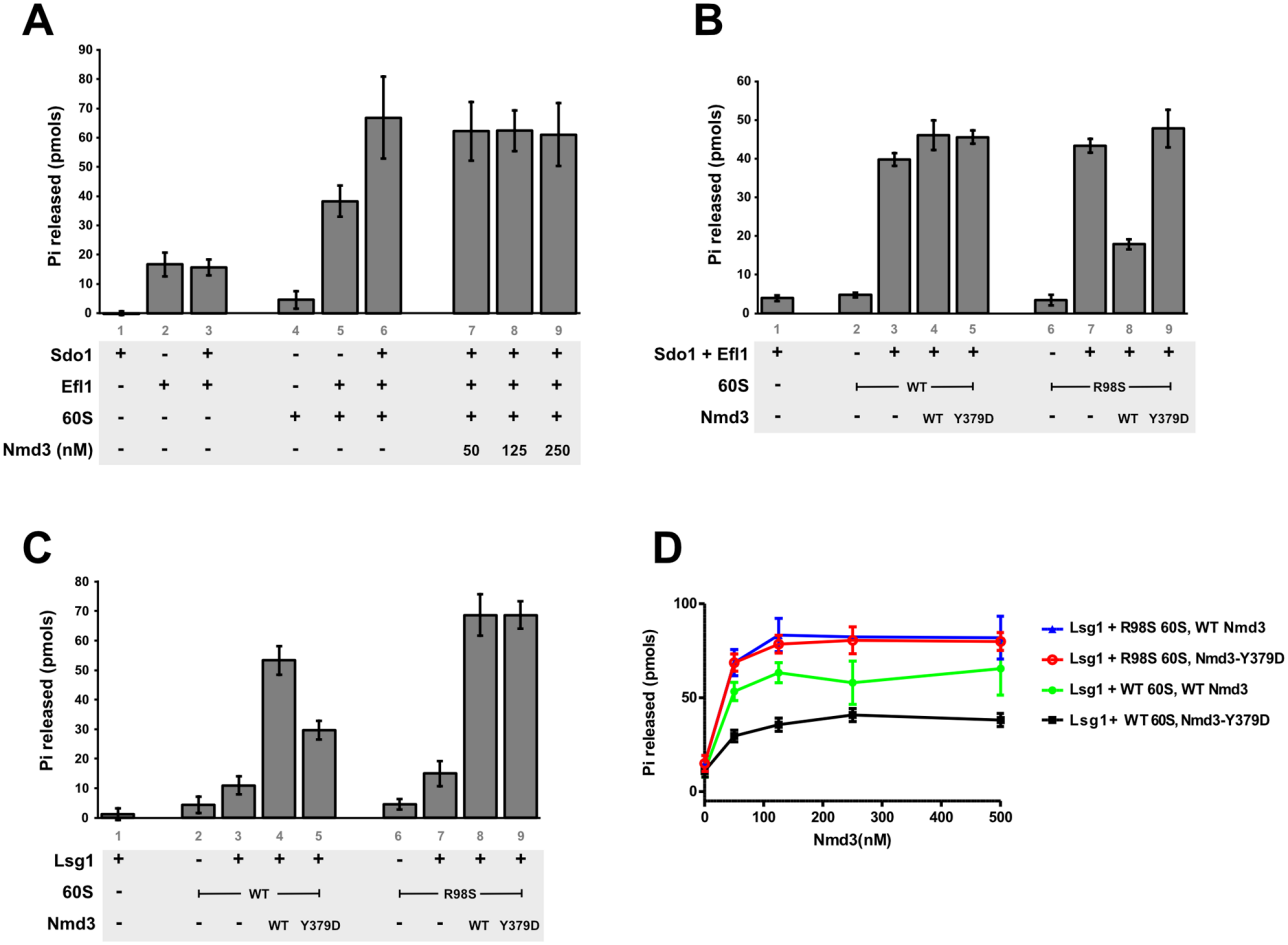
Efl1 activation is inhibited by Nmd3 on *rpl10-R98S* subunits. **A-B)** Sdo1 stimulated, 60S-dependent Efl1 GTPase activity was monitored by the release of free phosphate in reactions containing the indicated combinations of 50nM 60S subunits (wild-type or *rpl10-R98S*), 50nM Efl1, 250nM Sdo1, and the indicated concentrations of Nmd3 in (A) or 250nM Nmd3 in (B). All experiments were done in triplicate, the mean and SD are given. **C-D)** Nmd3 stimulated, 60S-dependent Lsg1 GTPase activity was monitored by the release of free phosphate in reactions containing the indicated combinations of 50nM 60S subunits (wild-type or *rpl10-R98S*), 250nM Lsg1, 250nM wild-type Nmd3 or Nmd3-Y379D in (C) or the indicated concentrations of wild-type Nmd3 or Nmd3-Y379D in (D). All experiments were done in triplicate, the mean and SD are given.

In our recent Nmd3-60S structural work, we observed Nmd3 engaged with the L1 stalk in multiple L1 stalk positions, ranging from partially open to fully closed [22]. In the fully closed position, the eL22-like domain of Nmd3 occupies the ribosomal P site in a position that is incompatible with Sdo1 binding in the P site. Nmd3 binding in the P site should therefore prevent the Sdo1-dependent activation of Efl1. We tested this idea *in vitro* with purified components. It has been shown previously that Sdo1 activates the GTPase activity of Efl1 in the presence of 60S subunits [33]. We used this assay to ask if Nmd3 would inhibit Sdo1-dependent Efl1 activity. We found that although Nmd3 binds to 60S subunits and activates Lsg1 [22], the presence of Nmd3 did not inhibit the Sdo1-dependent activation of Efl1 (Fig 5A). We were unable to test the effect of Tif6 stabilization of Nmd3 because in our hands the addition of Tif6 completely inhibits Sdo1-dependent Efl1 GTPase activity (S3 Fig).

The *rpl10-R98S* mutant blocks both Tif6 and Nmd3 release *in vivo*, and genetic suppression of this T-ALL associated mutant showed that mutations in Nmd3 restored Tif6 release. In addition, *rpl10-R98S* suppressing mutations alter the interaction of Nmd3 with 60S subunits, possibly destabilizing Nmd3 binding in the P site. Based on these observations, we considered that the Tif6 release defect in *rpl10-R98S* mutant ribosomes might be attributed to stabilization of Nmd3 in the P site, thereby blocking Sdo1 binding. We reasoned that the suppressing mutations identified in *NMD3* could destabilize Nmd3 from the fully engaged state in the P site. We found that whereas the addition of Nmd3 to wild-type subunits had no effect on Sdo1-dependent activation of Efl1, the addition of Nmd3 to *rpl10-R98S* subunits inhibited Sdo1-dependent activation of Efl1 GTPase (Fig 5B, compare lanes 3 and 4 to lanes 7 and 8). In contrast, the addition of Nmd3-Y379D, which suppresses the *rpl10-R98S* mutant *in vivo*, restored Sdo1-dependent activation of Efl1 GTPase in the presence of *rpl10-R98S* subunits (Fig 5B, lane 9). Thus, a mutation that alters the interaction of Nmd3 with the subunit overcomes the defect in Efl1 activation. These results strongly suggest that the molecular defect of the T-ALL-associated *rpl10-R98S* mutation is stabilization of Nmd3 in the P site where it blocks productive binding of Sdo1.

**Fig 6:**
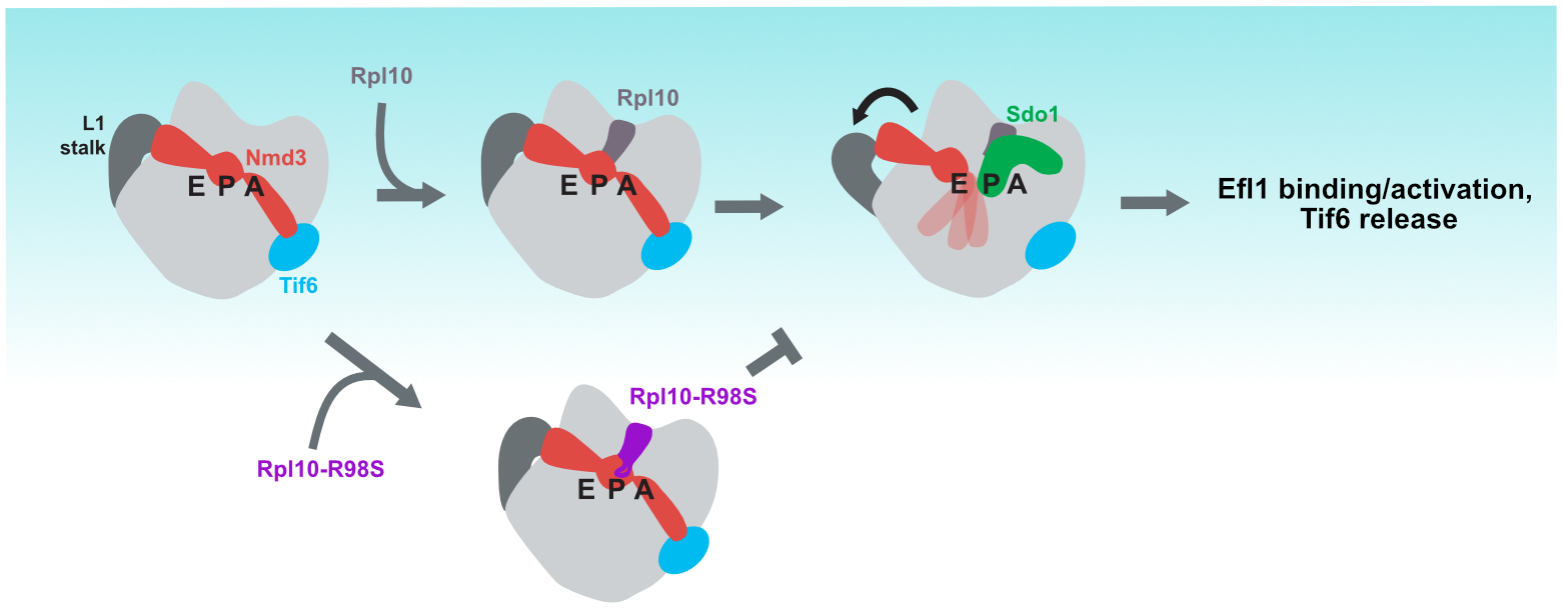
Model. In the fully closed L1 stalk position, the eIF5A domain of Nmd3 is engaged with the E site, while the eL22-like domain occupies the P site. This position is stabilized by the interaction between the N-terminus of Nmd3 with Tif6. We propose that, following Rpl10 loading, the linkage between Nmd3 and Tif6 is broken, destabilizing the N-terminus of Nmd3 and allowing Nmd3 retraction from the P site and Sdo1 binding.

We considered the possibility that the Nmd3-Y379D mutant failed to inhibit Efl1 activity due to a weakened affinity for 60S subunits. To test this, we used an *in vitro* assay for Nmd3-dependent Lsg1 activation. Because we previously showed that Nmd3 binding to 60S subunits was required for Lsg1 GTPase activation [22], we used Lsg1 activation as a proxy to test Nmd3 binding. We compared Lsg1 activation in the presence of either wild-type 60S subunits or *rpl10-R98S* 60S subunits and either wild-type Nmd3 or Nmd3-Y379D. We found that wild-type Nmd3 stimulated Lsg1 GTPase on both wild-type and *rpl10-R98S* 60S subunits (Fig 5C, lanes 4 and 8, Fig 6D). Interestingly, *rpl10-R98S* subunits supported slightly higher stimulation of Lsg1 GTPase activity compared to wild-type subunits. A similar increase in Lsg1 activity was previously observed in the presence of Tif6, which stabilizes Nmd3 in the fully engaged conformation where the eL22-like domain occupies the P site [22]. Thus, the increase in Lsg1 activation with *rpl10-R98S* subunits provides additional evidence that *rpl10-R98S* subunits trap Nmd3 in the fully engaged conformation. In contrast to wild-type Nmd3, Nmd3-Y379D supported only a low level of Lsg1 activation in the presence of wild-type 60S (Fig 5C, compare lanes 4 and 5). If Nmd3-Y379D simply had a reduced affinity for subunits, we would expect the reduced Lsg1 activity to be overcome at higher concentrations of Nmd3-Y379D, where more Nmd3 protein would be bound. However, Lsg1 activity was not recovered by increasing the concentration of Nmd3 (Fig 5D). Because the eIF5A domain of Nmd3 can bind to the L1 stalk independently of the eL22 domain binding in the P site, increasing concentrations of Nmd3 may saturate binding to the ribosome via the L1 stalk without increasing binding in the P site, explaining the lower activation of Lsg1 by Nmd3-Y379D on wild-type ribosomes. Interestingly, in the presence of *rpl10-R98S* subunits, Nmd3-Y379D stimulated Lsg1 to a greater extent than that observed for wild-type Nmd3 with wild-type 60S subunits (Fig 5C and 5D). This suggests that *rpl10-R98S* subunits might stabilize both wild-type and mutant Nmd3 in the P site, but Nmd3-Y379D can be repositioned in the presence of Sdo1. Together, these results argue strongly that the T-ALL-associated mutation *rpl10-R98S* traps Nmd3 in the P site. Mutations in Nmd3 that suppress *rpl10-R98S* weaken the interaction of Nmd3 with the P site, restoring the ability of Nmd3 to be retracted from the P site in the presence of Sdo1.

## Discussion

Mutations in the ribosomal protein Rpl10 (uL16) can be drivers of T-ALL [40]. We previously showed that these T-ALL mutations, including *rpl10-R98S*, disrupt late steps in cytoplasmic maturation of the 60S subunit, preventing the release of Nmd3 and Tif6 [40]. However, in that work we did not identify the molecular mechanism that led to the block in Tif6 and Nmd3 release. Because genetic analysis had placed the release of Nmd3 after the release of Tif6 [28], we expected that mutations that enhanced the release of Tif6 would suppress *rpl10-R98S*. Indeed, we had shown that one such mutant, *TIF6-V192F*, suppressed some mutations within the P site loop of Rpl10 (*rpl10-S104D*). Surprisingly, we found here that *TIF6-V192F* could not suppress *rpl10-R98S*. Instead, we found that mutations that enhanced the release of Nmd3 could suppress *rpl10-R98S*. This was difficult to reconcile with our earlier genetic analysis. However, recent atomic structures of Nmd3 on the 60S subunit revealed that Nmd3 spans the joining face of the 60S subunit, interacting with Rpl1 (uL1) of the L1 stalk, stretching through the E and P sites, and interacting directly with Tif6 [22,23]. Notably, the domain of Nmd3 in the P site would sterically block Sdo1 binding and prevent Tif6 release. Based on this structure, we proposed that Nmd3 interaction with Tif6 must be broken to permit the retraction of Nmd3 from the P site by the opening of the L1 stalk [22]. This retraction would allow Sdo1 to bind in the P site to trigger the release of Tif6. Thus, a model emerged that the defect of *rpl10-R98S* may be a failure in retraction of Nmd3 from the P site (Fig 6).

The eL22-like domain of Nmd3 contains an extended loop that projects into the mouth of the polypeptide exit tunnel. Because the P site loop of Rpl10 and the loop of Nmd3 occupy a similar position, it was recently suggested that the loading of Rpl10 could potentially influence Nmd3 release [23]. Arginine 98 is at the base of the Rpl10 loop and interacts with the sugar phosphate backbone of residues 1126 and 1127 of 25S rRNA. Loss of this interaction is expected to destabilize the P site loop and alter the interaction between Nmd3 and Rpl10. Although this could, in principle, affect the loading of Rpl10, our results indicate that the R98S mutation prevents the retraction of Nmd3 from the P site rather than the loading of Rpl10. This model of *rpl10-R98S* function is strongly supported by the genetic and biochemical results reported here.

We identified mutations in *NMD3* and *TIF6* that were extragenic suppressors of *rpl10-R98S*. These mutations mapped to the interface between Nmd3 and the 60S subunit and between Nmd3 and Tif6 and altered the interaction of Nmd3 with the 60S subunit. In addition, mutations in Nmd3 engineered to specifically disrupt hydrogen bonding with eL42 and rRNA also suppressed *rpl10-R98S*. Thus, mutations that destabilized Nmd3 on the pre-60S subunit suppressed *rpl10-R98S*. From a genetic perspective, it may seem counterintuitive that mutations that weaken the affinity of Nmd3 for the ribosome can act as dominant suppressors. However, their dominant nature can be readily explained. For example, wild-type Nmd3 will become trapped on pre-60S subunits in the cytosol, preventing entry of those 60S subunits into the translating pool but also sequester wild-type Nmd3 on the stalled subunits. Mutant Nmd3 that can be released will recycle to the nucleus unimpeded and support ribosome production.

We were also able to recapitulate the genetic effects of *rpl10-R98S* and suppression by mutations in Nmd3 with a reconstituted system of purified components. For this work, we used the activation of Efl1 by Sdo1 as a proxy for productive Sdo1 binding. Whereas wild-type Nmd3 did not compete with Sdo1 for binding in the P site on wild-type ribosomes, Nmd3 inhibited Sdo1 binding in the presence of *rpl10-R98S* ribosomes. Furthermore, this inhibition was reversed by an Nmd3 mutant, Nmd3-Y379D, that suppresses *rpl10-R98S* mutant cells *in vivo*. These results identify the defect of *rpl10-R98S*-containing ribosomes as a failure to release Nmd3 from the P site, and show that suppressing mutations bypass the entrapment of Nmd3 by destabilizing interactions of Nmd3 with the 60S subunit.

We proposed previously that Nmd3 is retracted from the P site by opening of the L1 stalk [22]. But whether Nmd3 release is coincident with retraction of the stalk or is a subsequent step remains an unanswered question. The persistence of Nmd3 binding to the L1 stalk could explain the observation that *lsg1* mutants, or the depletion of Lsg1, prevent recycling of Nmd3 but not Tif6 [22,31], leading to the conclusion that Nmd3 is released after Tif6. Although our results cannot definitively distinguish between these two models, several observations lead us to favor the latter. First, single particle reconstruction of the Nmd3-60S complex revealed Nmd3 bound to the L1-stalk in multiple conformations from partially open to fully closed, suggesting that Nmd3 interaction with Rpl1 is maintained independently of other interactions on the subunit [22]. And second, Nmd3-Y379D, which bypassed the inhibition of Efl1 activation on *rpl10-R98S* subunits, could efficiently stimulate Lsg1 GTPase on *rpl10-R98S* subunits. However, in the presence of wild-type 60S subunits, Nmd3-Y379D failed to activate Lsg1 to the same degree as did wild-type Nmd3, regardless of the concentration of protein. If Nmd3-Y379D simply had lower affinity for 60S, we would expect the defect in Lsg1 activation to be overcome at higher concentrations of Nmd3-Y379D. That we did not observe this is consistent with the interpretation that Nmd3-Y379D binding to the L1 stalk could be saturated, but its lower affinity for the P site prevented maximal activation of Lsg1. Thus, the Y379D mutation preferentially affects Nmd3 binding in the P site relative to overall subunit binding.

Thus far, our results imply a fairly simple model for suppression of *rpl10-R98S*—weakened interaction of Nmd3 for the subunit overcomes the retention of Nmd3 in the P site. This can be accomplished by mutations in Nmd3 or Tif6, all of which appear to reduce the affinity of Nmd3 for the ribosome. However, we would also expect mutations that weaken the affinity of Tif6 for the ribosome, such as *TIF6-V192F*, to suppress *rpl10-R98S*. That they do not suppress suggests additional subtleties to the mechanism of Nmd3 release. One interpretation is that bypass of *rpl10-R98S* requires stable association of Tif6 with the subunit. Although Efl1 does not appear to be required for the release of Nmd3 from wild-type subunits, it may assist in dislodging Nmd3 from the P site in *rpl10-R98S* mutant subunits.

The molecular defect of *rpl10-R98S* revealed in this work in yeast is likely relevant to human ribosomes in T-ALL patient cells. This conclusion is based on the high degree of conservation of Nmd3 and Rpl10—both human proteins can replace their yeast counterpart [12,46] and the amino acid sequence of Rpl10/uL16 is highly conserved across eukaryotes, with amino acid 98 invariantly an arginine. In addition, *rpl10-R98S* patients express only mutant *RPL10* in the mutated cells, and *rpl10-R98S* inhibited ribosome biogenesis in mammalian cells, as we have observed in yeast [40]. However, how this defect in ribosome biogenesis ultimately promotes T-ALL is not understood.

We previously demonstrated that the *rpl10-R98S* mutation had a severe impact on yeast ribosomes at both the structural and functional level, and proposed that *rpl10-R98S* promotes defective translational fidelity [42]. Within the context of T-ALL, we suspect that *rpl10-R98S* is a mutation arising early in disease pathogenesis. Whereas translation fidelity defects might support disease development by shifting the spectrum of expressed proteins toward a more oncogenic profile, the ribosome biogenesis defect and resulting lack of functional ribosomes is probably not beneficial for pre-leukemic cells, likely imposing high pressure on these cells to acquire additional mutations that suppress the biogenesis defect. While suppression would reverse the proliferation defect, it may come at the risk of allowing defective ribosomes to be used in translation. So far, the nature of suppressor mutations acquired in *rpl10-R98S* positive T-ALLs is unclear. Mutations in NMD3 and eIF6 (human Tif6) have not been detected in human *rpl10-R98S* positive T-ALL cases [42,47], suggesting that alternative suppressor mutations occur in the human patients. Thus, a current challenge is to identify and characterize *rpl10-R98S* suppressing mutations in T-ALL positive cells.

## Materials and methods

### Strains and plasmids

Yeast strains and plasmids used in this work are listed in Tables A and B of S1 Text. Oligos used in this work are listed in Table C of S1 Text. All cells were grown at 30°C in rich media (yeast extract and peptone) or appropriate synthetic drop-out medium with 2% glucose or 1% galactose as the carbon source. Strains AJY2781 and AJY2784 were made by introducing plasmid pAJ2522 or pAJ2726 into the *rpl10* deletion strain AJY1437 [32] by plasmid shuffle. Spontaneous suppressing mutations were identified in the AJY2784 background, including *NMD3-Y379D* (AJY2846) and *TIF6-G189V* (AJY2848). AJY2781, AJY2784, AJY2846, and AJY2848 were used to generate AJY3937, AJY3938, AJY3939, and AJY3940, respectively: pDEGQ2 was introduced into each strain, and the *TIF6-GFP*::*His3MX* cassette (amplified from *TIF6-GFP* cells (Open Biosystems) using AJO454 and AJO1384) was integrated into the genome by homologous recombination. AJY3909 was made by crossing AJY2846 and AJY1837 [31] and AJY3943 was made by crossing AJY2848 with AJY1958. Strain AJY1958 was made by crossing relevant mutants. To make AJY3941, the *TIF6* locus was amplified in two parts from *TIF6-GFP* strain (Open Biosystems) genomic DNA, using mutagenic oligos to introduce the V192F mutation. The 5’ half of *TIF6-GFP* was amplified using AJO453 and AJO933, and the remaining half of was amplified with AJO932 and AJO454, such that the two PCR products overlapped. PCR products derived from these reactions were used in a final reaction to make the full length *TIF6-V192F-GFP* PCR product, which was integrated into the genome of AJY3901(NatMX::P_*GAL*_-*RPL10 Δtif6*::*KanMX)* by homologous recombination. AJY3249 was made by amplifying the His3MX-PGAL1-3HA cassette [48] with homology to the *NMD3* locus using AJO2031 and AJO2032, integrating into BY4743 (Open Biosystems), followed by sporulation and dissection.

To make pAJ2805, *NMD3-Y379D* was gap-rescued from the genome of AJY2846 using pAJ409 digested with SnaBI and HpaI. *NMD3-Y379D* was amplified from pAJ2805 using oligos AJO360 and AJO2035, digested with NdeI and SpeI restriction enzymes, and ligated into the same sites in pAJ2392. pAJ3609 was generated by inverse PCR of pAJ409 using oligos AJO2558 and AJO2554. Similarly, pAJ3581 was made by inverse PCR of pAJ409 using AJO2704 and AJO2705. pAJ729 was made by amplifying the *TIF6* locus from genomic DNA using oligos AJO453 and AJO454. PCR product was digested with SalI and HindIII and inserted into the same sites in pRS426. pAJ2846 was made by digesting *TIF6* sequence from pAJ2665 [32] with BamHI and XhoI and ligating into the same sites in pRS415. pAJ2828 was made by inverse PCR using oligos AJO1820 and AJO1821 with pAJ2665 as template. To make pAJ2833, the *TIF6-G189V* sequence was digested from pAJ2828 using SstI and XhoI enzymes, and ligated into the same sites in pRS415. pAJ3023 was made by digesting the *TIF6* allele from pAJ2828 using XhoI and SacI enzymes, and ligating into the same sites in pRS426. pAJ3024 was generated by digesting *TIF6-V192F* from pAJ2240 [32] using XhoI and BamHI enzymes, and ligating into the same sites in pRS426. pAJ2839 and pAJ2840 were made by digesting *RPL10* from pAJ2522/pAJ2726 using SacI and BamHI, and ligating into the same sites in pRS313. To make pAJ2982, genomic DNA was amplified using oligos AJO1413 and AJO1414, digested with NdeI and XhoI restriction enzymes, and inserted into the same sites in pET21a. The kemptide tag was added by inverse PCR using AJO1762 and AJO1763. To make pAJ3114, an internal 8HIS tag was integrated between *EFL1* amino acids 458 and 459 in a pET21d vector containing *EFL1* with its natural C-terminus.

### Identification of spontaneous suppressors

Spontaneous suppressors of *rpl10-R98S* were identified in AJY2784. Individual slow-growing colonies were cultured independently and passaged several times before plating. After streaking for single colonies, large ‘fast-growing’ colonies were isolated and genomic DNA was prepared. The *RPL10* allele was amplified (using AJO264 and AJO268) and sequenced to confirm that cells had not reverted to WT. The *NMD3* allele was amplified (using AJO238 and AJO329) and sequenced to identify *NMD3* suppressing alleles. The identified *NMD3- Y379D* suppressor was saved as strain AJY2846. The *TIF6-G189V* mutant was identified by high-throughput SOLiD sequencing of gDNA from a suppressor isolate, saved as strain AJY2848. Sequencing and SNP analysis was carried out by the Genome Sequencing and Analysis Facility at the University of Texas at Austin.

### *TIF6* mutagenesis

The open reading frame of *TIF6* was randomly mutagenized by PCR using Taq DNA polymerase and oligos AJO453 and AJO454, with wild-type *TIF6* plasmid pAJ2846 as template. PCR product was then co-transformed with gapped starting vector, to allow recombination of the mutagenized PCR products back into the starting vector, in AJY3901. Fast growing colonies were selected and *TIF6*-containing plasmids were extracted and sequenced. *TIF6* mutant plasmids were then re-introduced into *rpl10-R98S* cells to confirm suppression.

### Microscopy

For direct fluorescence experiments, cells were grown to saturation in selective medium containing galactose, then diluted back 20-fold in medium containing 2% glucose and grown for an additional hour to repress the expression of genomic *RPL10*. Images were captured using a Nikon E800 microscope fitted with a 100x Plan Apo objective and a Photometrics CoolSNAP ES camera controlled by NIS- Elements software. For indirect immunofluorescence, cells were grown in selective medium containing galactose before adding glucose to 2% to repress the expression of wild-type genomic *RPL10* for 2h. LMB was added to a final concentration of 0.1 μg/ml for 15 min to block Nmd3 shuttling. Cells were fixed with a 1:9 volume of 37% formaldehyde for 40min, then washed with Ksorb buffer (0.1 M potassium phosphate [pH 6.6], 1.2 M sorbitol). Cells were permeabilized in cold methanol followed by washing in acetone. Anti-Nmd3 antibody [49] was diluted 3,000 fold in PBS with 0.1% BSA. Cy3-conjugated donkey anti—rabbit antibody (Jackson ImmunoResearch Laboratories, Inc.) was used at a 300-fold dilution. After antibody application, cells were incubated for 1 min in 1 μg/ml DAPI and mounted in Aqua-Poly/Mount.

### Sucrose density gradient sedimentation and polysome profile analysis

For polysome profile analysis, cells were initially grown to saturation in the presence of galactose then diluted back to 3x10^6^ cells/ml in glucose-containing media, and growth was continued until cultures reached 1.2x10^7^ cells/ml. 100μg/ml cycloheximide (CHX) was added to each culture, followed by incubation for an addition 10min at 30°C. Cultures were then immediately poured over ice and harvested by centrifugation. To monitor Nmd3 sedimentation, cells were grown only in glucose-containing selective media. Liquid cultures were grown to mid-log phase, treated with CHX and harvested as previously described. Cell extracts were prepared at 0–4°C: Cells were washed with lysis buffer (100mM KCl, 50mM Tris-HCl pH 7.5, 5mM MgCl2, 100μg/ml CHX, 6mM beta-mercaptoethanol (βME), 1mM PMSF, 1μg/ml leupeptin, 1μg/ml pepstatin A), and lysed by vortexing in the presence of glass beads. Extracts were clarified by centrifugation for 10 min at 15,000 g at 4°C, and 9A_260_ units of clarified extract were loaded onto 7–47% sucrose gradients, prepared in lysis buffer, and centrifuged for 2.5 hours at 40,000 rpm (Beckman SW40). Gradients were fractionated (ISCO Model 640) with continuous monitoring at 254nm. To monitor Nmd3 sedimentation, fractions were precipitated with 100% EtOH overnight at -20°C, then centrifuged for 30min, 15,000 g at 4°C. Pellets were resuspended in Laemmli buffer and boiled at 99°C for 3 min. Proteins were separated on 10% SDS-PAGE gels, transferred to a nitrocellulose membrane, and subjected to Western blot analysis using anti-Nmd3 [49] and anti-Rpl8 (from K. Lo) antibodies.

### GTPase assays

Specified amounts of Efl1, Sdo1, Lsg1, Nmd3, and 60S, as indicated in the figure legends, were mixed in a 20ul volume in 1x GTPase buffer (20mM HEPES-KOH, pH 7.4, 2mM MgOAc, 50mM KOAc, 1mMDTT) containing 25uM GTP. Reactions were spiked with approximately 1x10^5^ cpm of [gamma -32P]-GTP to trace the hydrolysis of gamma phosphate. Reactions were incubated at 30°C for 10 minutes and stopped by addition of 5ul of 0.5M EDTA. 1ul of each reaction was spotted on a TLC plate (PEI-cellulose, Sigma-Aldrich). Free phosphate was separated from GTP by developing the TLC plate for 10 minutes in 0.8M LiCl, 0.8M CH_3_COOH. Plates were imaged on a phosphoimager screen and signal intensities for free phosphate and GTP were analyzed using ImageJ software. All samples were corrected for non-enzymatic background hydrolysis. Reactions containing Nmd3 were corrected for background hydrolysis by free Nmd3 protein. Percent GTP hydrolysis was calculated as (free phosphate/total phosphate) and picomoles of phosphate release were calculated as Percent GTP hydrolysis*[GTP]. Picomole phosphate release values from Nmd3 titration assays were fitted to saturating-specific single site binding curves using Graphpad Prizm software.

### Protein purification

**Lsg1-6His**: Yeast Lsg1 protein with a C-terminal 6xHis tag was purified from Codon plus (RIL) *E*. *coli* cells (Stratagene) with pAJ3420 as described in [22].

**MBP-TEV-His6-Nmd3**: For WT Nmd3 1 liter of BJ5464 with pAJ1381 was grown to OD_600_ of 0.6 in selective medium containing 2% glucose. For Nmd3-Y379D, 25 ml overnight culture of BJ5464 with pAJ2849 grown in selective medium with 2% glucose was diluted to OD_600_ = 0.03 in 100ml of same medium and grown for 8–10 hours, then diluted to OD_600_ = 0.03 in 500ml of selective medium with 1% raffinose and grown overnight at 30°C. The culture was then induced at OD_600_ = 0.4 by adding galactose to 1% final concentration. Both WT and mutant proteins were purified using the same method: cells were harvested, washed, and resuspended in two volumes of extract buffer (50mM Tris, pH 8, 450mM NaCl, 100 mM KCl, 10% glycerol, 1mM PMSF, 1μg/ml leupeptin, 1μg/ml pepstatin A). Cells were disrupted by vortexing with glass beads and crude extract was clarified by centrifugation at 4°C, first for 10 min at 10,000 *g* and then for 20 minutes at 25,000 *g*. Imidazole was added to 10mM and clarified lysate was incubated with 0.5ml Ni-NTA Agarose beads (Invitrogen, R901-15) equilibrated in extract buffer for 1 hour at 4°C. The beads were washed twice with 10ml of extract buffer containing 10mM and 20mM imidazole respectively and the protein was eluted in 0.25ml fractions of extract buffer supplemented with 250mM imidazole. Fractions containing protein were pooled and diluted in 9 volumes of Q buffer (40mM Tris-HCl, pH 7.0, 10% glycerol and 1mM DTT). Bound proteins were eluted with a 20ml linear NaCl gradient (50mM to 1M) in Q buffer. Fractions containing Nmd3 were pooled and dialyzed in 20mM Tris, pH7.5, 150mM KOAc, 1mM DTT and 10% glycerol. Dialyzed protein was stored at -80°C.

**Efl1-internal His8**: Yeast Efl1 protein with an internal 8xHis tag in domain II of the protein was purified from Codon plus (RIL) *E*. *coli* cells (Stratagene) with pAJ3114. 1 liter of bacterial culture was grown to OD_600_ of 0.5 and induced with 1mM IPTG for 4 hours at 30°C. Cells were harvested and washed with Lysis buffer (40mM Tris, pH 8.0, 500mM NaCl and 10% glycerol). The cell pellet was resuspended in 40ml Lysis buffer (supplemented with 5mM βME, 1mM PMSF and 1μM each leupeptin and pepstatin) and disrupted by sonication. Lysates were cleared by centrifugation at 20,000 g for 20 minutes. Imidazole was added to 10mM. Clarified lysate was bound to a 1ml Ni-NTA column (HisTrap HP, GE Healthcare) by pumping at 1ml/min. The column was first washed with 10ml lysis buffer supplemented with 10mM imidazole and 5mM βME and then with low salt buffer (40mM Tris, pH 8.0, 50mM NaCl, 10% glycerol, 20mM imidazole and 5mM βME). Bound protein was eluted with low salt buffer with 125mM imidazole. Fractions containing protein were pooled and bound to 2ml Source Q (GE Healthcare) column pre-equilibrated with buffer A (100mM Tris pH 8.0, 50mM NaCl, 2mM DTT and 0.2mM EDTA). Column was washed with 10ml buffer A. Bound proteins were eluted with a 20ml linear NaCl gradient (50mM to 1M) in buffer A. Fractions containing Efl1 were pooled and dialyzed in 20mM Tris, pH 7.5, 150mM KOAc, 1mM DTT and 10% glycerol. Dialyzed protein was stored at -80°C.

**Sdo1-kemptide-6His**: Yeast Sdo1 protein with C-terminal kemptide and 6xHis tag was purified from Codon plus (RIL) *E*. *coli* cells (Stratagene) with pAJ2982. 1 liter of bacterial culture was grown to OD_600_ of 0.5 and induced with 1mM IPTG for 4 hours at 30°C. Cells were harvested and washed with Lysis buffer (40mM Tris, pH 8.0, 500mM NaCl and 10% glycerol). The cell pellet was resuspended in 40ml Lysis buffer (supplemented with 5mM βME, 1mM PMSF and 1μM each leupeptin and pepstatin) and disrupted by sonication. Lysates were cleared by centrifugation at 20,000 g for 20 minutes. Imidazole was added to 10mM. Clarified lysate was bound to a 2 ml Ni-NTA column (HisTrap HP, GE Healthcare) by pumping at 1ml/min. The column was first washed with 20ml lysis buffer supplemented with 10mM imidazole and 5mM βME and then with 20ml lysis buffer supplemented with 30mM imidazole and 5mM βME. Bound protein was eluted using lysis buffer with 250mM imidazole in 0.67ml fractions. Fractions containing Sdo1 protein were pooled and loaded on 150ml Sephacryl S-200 (Pharmacia) gel filtration column pre-equilibrated in S-200 buffer (40mM Tris-HCl pH 8.0, 150mM NaCl, 10% glycerol, 5mM βME). Bound protein was eluted in S-200 buffer. Fractions containing Sdo1 were pooled and dialyzed in 20mM Tris, pH 7.5, 150mM KOAc, 1mM DTT and 10% glycerol. Dialyzed protein was stored at -80°C.

### Purification of 60S subunits from yeast

Yeast strains AJY2781 and AJY2846 were grown in 2L of YPD to OD600 of 1.0. Cells were transferred to ice for 1h before harvesting by centrifugation at 4°C. Cell pellets were washed and resuspended in 6ml of binding buffer (20mM HEPES-KOH, pH 7.6, 60mM NH_4_Cl, 5mM Mg(oAc)_2_, 2mM DTT, 1mM PMSF). Cells were disrupted by glass bead lysis and cleared by centrifugation at 30,000 g for 25 min at 4°C. Active 80S ribosomes were purified from cell extracts using cysteine-charged sulfolink columns as described [41]. To dissociate subunits, ribosome pellets were resuspended in 1ml elution buffer (20mM HEPES-KOH, pH 7.6, 60mM NH_4_Cl, 500mM KCl, 10mM Mg(oAc)_2_, 2mM DTT, 1mM PMSF and 1uM each leupeptin and pepstatin) and incubated at 37°C for 30 min after the addition of puromycin and GTP, each to 1mM final concentration. The sample was then centrifuged through 10–30% sucrose gradients in elution buffer for 4h at 32,000 rpm in an SW40 rotor (Beckman Coulter). Fractions containing the 60S and 40S peaks were pooled separately and concentrated using an Amicon Ultra-4 50 K (Millipore), and buffer was changed to Ribosome storage buffer (20mM HEPES-KOH, pH 7.6, 100mM KCl, 5mM Mg(oAc)_2_, 2mM DTT, 250mM sucrose).

## Supporting information

S1 Fig Complementation of *NMD3* mutants. 10-fold serial dilutions of AJY3249 (P_GAL_*-NMD3*) cells with empty vector, or vector containing either WT (pAJ409) or mutant *NMD3* (pAJ2805 or pAJ3609). Cells were plated on galactose-containing media (left) to allow expression of WT *NMD3* from the genome and compared to cells plated on glucose-containing media (right) to shut down expression of genomic *NMD3*.
(TIFF)

S2 Fig Complementation of *TIF6* mutants. 10-fold serial dilutions of AJY1700 (tif6Δ with *P*_*GAL10*_*-TIF6-myc URA3* plasmid) cells with empty vector or vector containing either wild-type (pAJ2846) or mutant *TIF6* (pAJ2833 or pAJ3401). Cells were grown on galactose-containing media (left) to allow expression of the *P*_*GAL10*_*-TIF6-myc URA3* plasmid. The *URA3* plasmid was shuffled out by plating cells on glucose-containing media with 5FOA (right).
(TIFF)

S3 Fig Tif6 inhibits Sdo1-stimulated Efl1 activation. Sdo1-stimulated, 60S-dependent Efl1 GTPase activity was monitored by the release of free phosphate in reactions containing the indicated combinations of 100nM 60S subunits, 50nM Efl1, 125nM Sdo1, and 125nM Tif6. Mean and SD values are reported for experiments repeated twice.
(TIFF)

S1 Table Table A, Strains used in this study. Table B, Plasmids used in this study. Table C, Oligos used in this study.
(DOCX)

S1 Data Supplemental data for Fig 2C. (CSV)

S2 Data Supplemental data for Fig 5A. (XLSX)

S3 Data Supplemental data for Fig 5B. (XLSX)

S4 Data Supplemental data for Fig 5C and 5D. (XLSX)

S5 Data Supplemental data for S3 Fig. (XLSX)
