# Supplementary figures and images for: The T-cell leukemia related rpl10-R98S mutant traps the 60S export adapter Nmd3 in the ribosomal P site in yeast
Source: PLoS Genet. 2017 Jul 17;13(7):e1006894. doi: 10.1371/journal.pgen.1006894 (PMC5536393; doi:10.1371/journal.pgen.1006894)

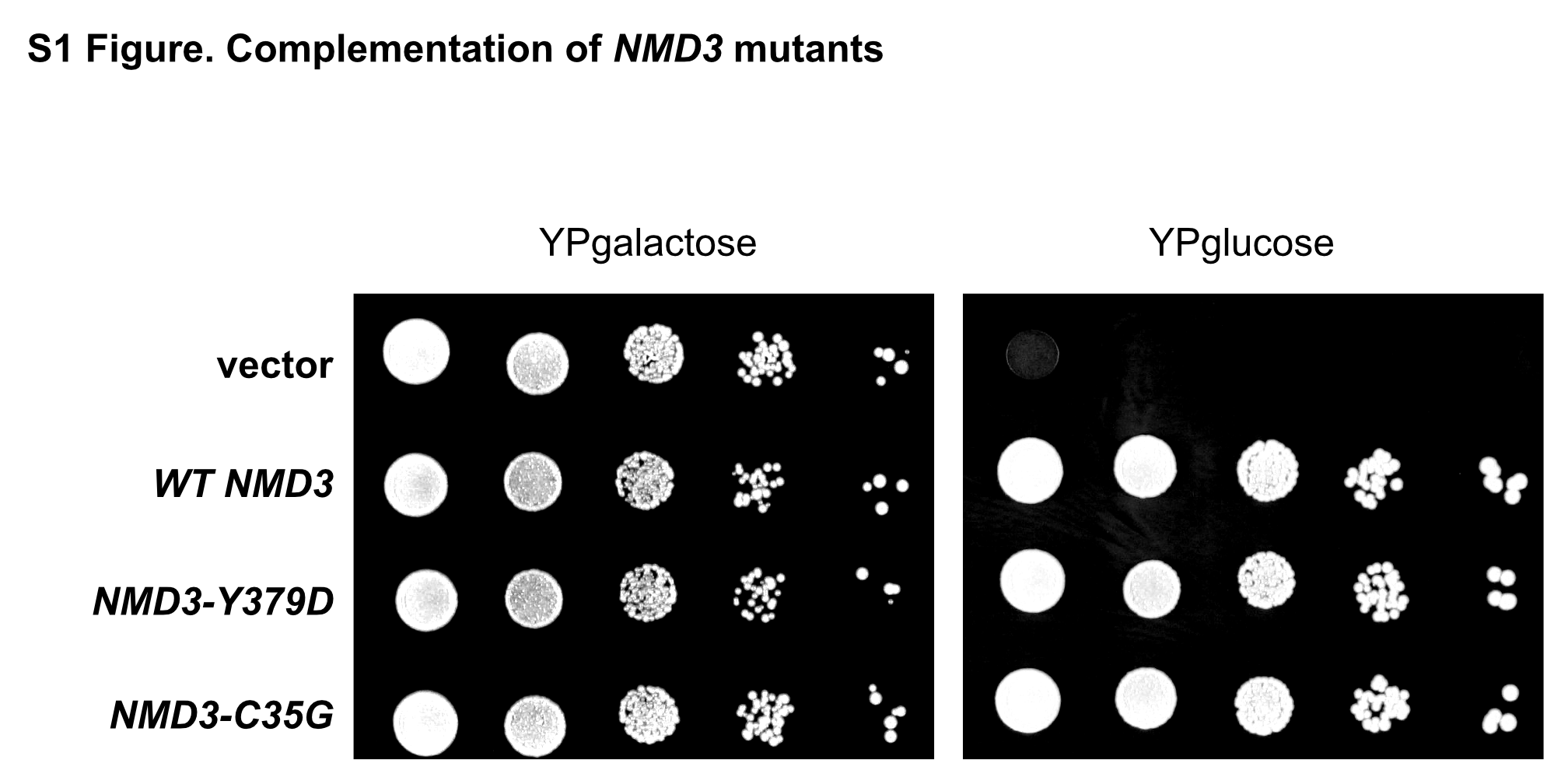

Supplement: S1 Fig — 10-fold serial dilutions of AJY3249 (PGAL-NMD3) cells with empty vector, or vector containing either WT (pAJ409) or mutant NMD3 (pAJ2805 or pAJ3609). Cells were plated on galactose-containing media (left) to allow expression of WT NMD3 from the genome and compared to cells plated on glucose-containing media (right) to shut down expression of genomic NMD3. (TIFF) [file pgen.1006894.s001.tiff]

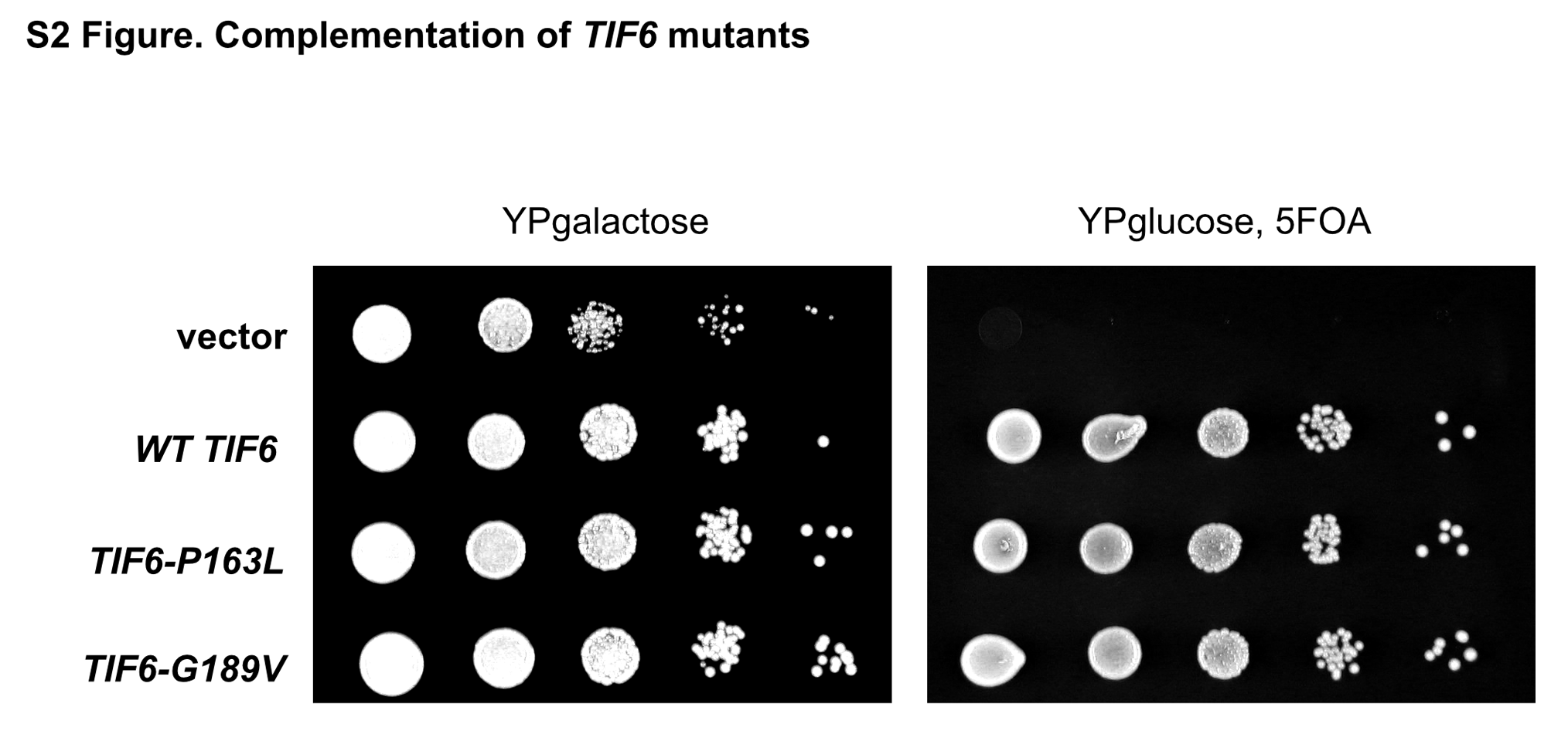

Supplement: S2 Fig — 10-fold serial dilutions of AJY1700 (tif6Δ with PGAL10-TIF6-myc URA3 plasmid) cells with empty vector or vector containing either wild-type (pAJ2846) or mutant TIF6 (pAJ2833 or pAJ3401). Cells were grown on galactose-containing media (left) to allow expression of the PGAL10-TIF6-myc URA3 plasmid. The URA3 plasmid was shuffled out by plating cells on glucose-containing media with 5FOA (right). (TIFF) [file pgen.1006894.s002.tiff]

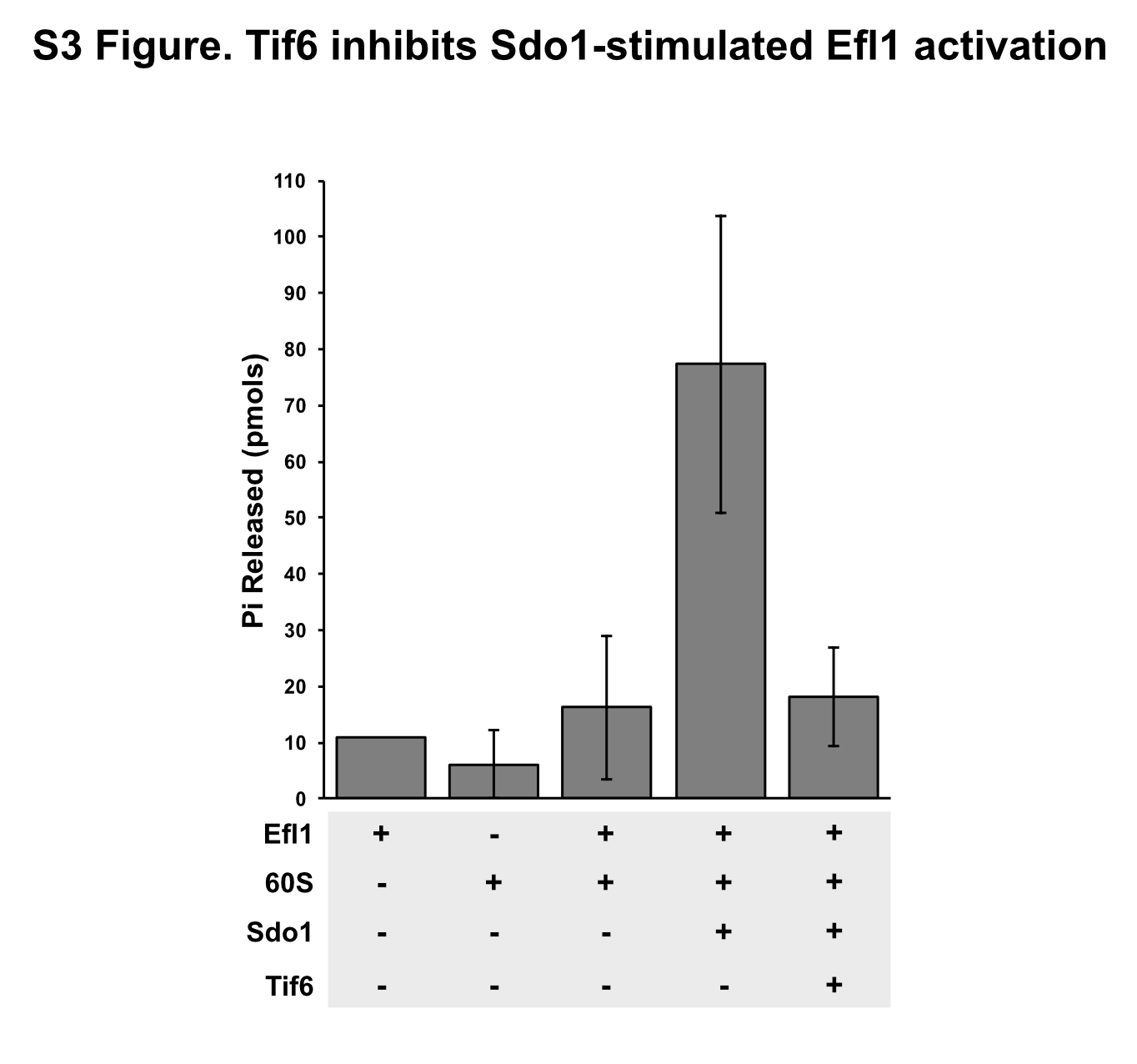

Supplement: S3 Fig — Sdo1-stimulated, 60S-dependent Efl1 GTPase activity was monitored by the release of free phosphate in reactions containing the indicated combinations of 100nM 60S subunits, 50nM Efl1, 125nM Sdo1, and 125nM Tif6. Mean and SD values are reported for experiments repeated twice. (TIFF) [file pgen.1006894.s003.tiff]
