# Supplementary material for: The T-cell leukemia related rpl10-R98S mutant traps the 60S export adapter Nmd3 in the ribosomal P site in yeast
Source: PLoS Genet. 2017 Jul 17;13(7):e1006894. doi: 10.1371/journal.pgen.1006894 (PMC5536393; doi:10.1371/journal.pgen.1006894)
Supplement: S1 Table — Table A, Strains used in this study. Table B, Plasmids used in this study. Table C, Oligos used in this study. (DOCX) [file pgen.1006894.s004.docx]

#### Table A. Strains used in this study

| **Strain** | **Genotype** | **Source** |
| --- | --- | --- |
| AJY1700 | *MATα tif6∆::KanMX with pAJ1194 (P_GAL10_-TIF6-myc URA3) his3∆1 leu2∆0 ura3∆0* | [1] |
| AJY1958 | *MATα rpl10∆::KanMX CRM1-T539C with* *pAJ730 (P_GAL10_-RPL10 URA) his3∆1 leu2∆0 ura3∆0* | This Study |
| AJY2766 | *MATα* *KanMX::P_GAL1_-RPL10 TIF6-GFP::HIS3 ade2 ade3 leu2∆0 ura3∆0 his3Δ1* | [2] |
| AJY2781 | *MATa rpl10∆::KanMX with pAJ2522 (RPL10 LEU2) his3Δ1 leu2Δ0 met15Δ0 ura3Δ0* | This Study |
| AJY2784 | *MATa rpl10∆::KanMX with pAJ2726 (rpl10-R98S LEU2) his3Δ1 leu2Δ0 met15Δ0 ura3Δ0* | This Study |
| AJY2846 | *MATa rpl10∆::KanMX NMD3-Y379D with pAJ2726 (rpl10-R98S LEU2) his3Δ1 leu2Δ0 met15Δ0 ura3Δ0* | This Study |
| AJY2848 | *MATa rpl10∆::KanMX TIF6-G189V with pAJ2726 (rpl10-R98S LEU2) his3Δ1 leu2Δ0 met15Δ0 ura3Δ0* | This Study |
| AJY2981 | *MATa KanMX::P_GAL1_-3xHA-EFL1 ade2-1 can1-100 his3-11,15 leu2-3,112 trp1-1 ura3-1* | [1] |
| AJY3249 | *MATα HIS3MX::P_GAL1_-3xHA-NMD3 his3Δ1 leu2Δ0 ura3Δ0* | This Study |
| AJY3373 | *MATα* *KanMX*::*P_GAL1_*-*RPL10 his3*Δ*1 leu2*Δ*0 ura3*Δ*0* | [3] |
| AJY3909 | *MATα* *rpl10∆::KanMX NMD3-Y379D crm1-T539C with* pAJ2726 *(rpl10-R98S LEU2),* pAJ730 *(P_GAL10_-RPL10 URA) leu2*Δ*0 ura3*Δ*0* | This Study |
| AJY3937 | *MAT*a *rpl10Δ::KanMX TIF6-GFP::HIS3MX with* pAJ730 (*P_GAL10_*-*RPL10 URA3),* pAJ2522 *(RPL10 LEU2) met15Δ0 his3*Δ*1 leu2Δ0 ura3Δ0* | This Study |
| AJY3938 | *MAT*a *rpl10Δ::KanMX TIF6-GFP::HIS3MX with* pAJ730 (*P_GAL10_*-*RPL10 URA3),* pAJ2726 *(rpl10-R98S LEU2) met15Δ0 his3*Δ*1 leu2Δ0 ura3Δ0* | This Study |
| AJY3939 | *MAT*a *rpl10Δ::KanMX NMD3-Y379D TIF6-GFP::HIS3MX with* pAJ730 (*P_GAL10_*-*RPL10 URA3),* pAJ2726 *(rpl10-R98S LEU2) met15Δ0 his3*Δ*1 leu2Δ0 ura3Δ0* | This Study |
| AJY3940 | *MAT*a *rpl10Δ::KanMX TIF6-G189V-GFP::HIS3MX with* pAJ730 (*P_GAL10_*-*RPL10 URA3),* pAJ2726 *(rpl10-R98S LEU2) met15Δ0 his3*Δ*1 leu2Δ0 ura3Δ0* | This Study |
| AJY3941 | *NatMX::P_GAL1_-RPL10 TIF6-V192F-GFP::HIS3 ade2 ura3 leu2* | This Study |
| AJY3943 | *rpl10∆::KanMX TIF6-G189V crm1-T539C with* pAJ2726 *(rpl10-R98S LEU2),* pAJ730 *(P_GAL10_-RPL10 URA) his3∆1 leu2∆0 ura3∆0* | This Study |
| BJ5464 | *MATα pep4::HIS3 prb1Δ1.6R can1 ura3-52 trp1 leu2Δ1 his3*Δ*200* | [4] |

#### Table B. Plasmids used in this study

| **Plasmid** | **Description** | **Source** |
| --- | --- | --- |
| pDEGQ2 | *GAL-RPL10 URA3* | [5] |
| pAJ409 | *NMD3 URA3 CEN* | [6] |
| pAJ729 | *TIF6 URA3 2u* | This Study |
| pAJ1381 | *P_GPD_-MBP-TEV-6HIS-NMD3 LEU2 2u* | [7] |
| pAJ2522 | *RPL10 LEU2 CEN* | [2] |
| pAJ2545 | *EFL1 HIS3 CEN* | [2] |
| pAJ2609 | *rpl10-S104D LEU2 CEN* | [2] |
| pAJ2664 | *EFL1-I678T HIS3 CEN* | [2] |
| pAJ2679 | *EFL1-linker mutant HIS3 CEN* | [2] |
| pAJ2726 | *rpl10-R98S LEU2 CEN* | [3] |
| pAJ2805 | *NMD3-Y379D URA3 CEN* | This Study |
| pAJ2833 | *TIF6-G189V LEU2 CEN* | This Study |
| pAJ2839 | *RPL10 HIS3 CEN* | This Study |
| pAJ2849 | *P_GAL_-MBP-TEV-6HIS-NMD3 LEU2 2u* | This Study |
| pAJ2840 | *rpl10-R98S HIS3 CEN* | This Study |
| pAJ2846 | *TIF6 LEU2 CEN* | This Study |
| pAJ2982 | *T7-SDO1-6HIS-kemptide Amp^R^* | This Study |
| pAJ3023 | *TIF6-G189V URA3 2u* | This Study |
| pAJ3024 | *TIF6-V192F URA3 2u* | This Study |
| pAJ3114 | *T7-EFL1-internal 8HIS Amp^R^* | This Study |
| pAJ3281 | *EFL1-A669G, S1028G HIS3 CEN* | [2] |
| pAJ3401 | *TIF6-P163L LEU2 CEN* | This Study |
| pAJ3420 | *LSG1-6HIS* | [8] |
| pAJ3581 | *NMD3-N378A, N380A URA3 CEN* | This Study |
| pAJ3609 | *NMD3-C35G URA3 CEN* | This Study |

#### Table C. Oligos used in this study

| **Oligo** | **Sequence** |
| --- | --- |
| AJO238 | 5’-TTTTATTTGTCGCCTGGTACAT-3’ |
| AJO264 | 5’-CGCGGATCCGAAACTAGTTAGCAC-3’ |
| AJO268 | 5’-CGCGGATCCTACCCAACATGCTGAAC-3’ |
| AJO329 | 5’-CTGCATCCAGTATACACACCCA-3’ |
| AJO360 | 5’-GGGCTTCATATGGAATTCACACCTATAGA-3’ |
| AJO453 | 5’-CGGAAGCTTGCATTCTGGACGAAATCC-3’ |
| AJO454 | 5’-GCTGTCGACTCTTTCGCATACAACTG-3’ |
| AJO932 | 5’-CCGTGGGAGCTCATTTGTCGGTGC-3’ |
| AJO933 | 5’-CGACAAATGAGCTCCCACGGTTAACG-3’ |
| AJO1384 | 5’-TGCTGGTACGCGTATCATCGG-3’ |
| AJO1413 | 5’-GCTCATATGCCTATCAATCAACCGTCG-3’ |
| AJO1414 | 5’-GCACTCGAGGTTATGCGTTGTATTATCTATG-3’ |
| AJO1762 | 5’-GTGGTGGTGGTGGTGGTGCTCGAGGTTATGCG-3’ |
| AJO1763 | 5’-CTGCGTCGCGCTAGCCTTGGTTGAGATCCGGCTGCTAACAAAGC-3’ |
| AJO1820 | 5’-GTTAGTTCAGTTGTCGGTGCCG-3’ |
| AJO1821 | 5’-ACGGTTAACGGTACCAGCCACC-3’ |
| AJO2031 | 5’-TGAGGAACCTTGGCTTTATCTTTTATTTGTCGCCTGGTACGAATTCGAGCTCGTTTAAAC-3’ |
| AJO2032 | 5’-CATTTTGGTGCTGGTGCGGATCTATAGGTGTGAATTCCATGCACTGAGCAGCGTAATCTG-3’ |
| AJO2035 | 5’-GCCTACTAGTGCGTGCCGGAAAGGGTGTCC-3’ |
| AJO2554 | 5’-GGTTATGACTGTATCAAACTGAC-3’ |
| AJO2558 | 5’-CATAACCAGCCCAGTGGAC-3’ |
| AJO2704 | 5’-TATGCCTCAGATCTTTTTGATGGGTTAAATATC-3’ |
| AJO2705 | 5’- TGCCGAGTTTGCAATGAAATAACCC-3’ |

**REFERENCES**

1. Lo K-Y, Li Z, Bussiere C, Bresson S, Marcotte EM, Johnson AW. Defining the pathway of cytoplasmic maturation of the 60S ribosomal subunit. Mol Cell. 2010;39: 196–208. doi:10.1016/j.molcel.2010.06.018

2. Bussiere C, Hashem Y, Arora S, Frank J, Johnson AW. Integrity of the P-site is probed during maturation of the 60S ribosomal subunit. J Cell Biol. Rockefeller Univ Press; 2012;197: 747–759. doi:10.1083/jcb.201112131

3. De Keersmaecker K, Atak ZK, Li N, Vicente C, Patchett S, Girardi T, et al. Exome sequencing identifies mutation in CNOT3 and ribosomal genes RPL5 and RPL10 in T-cell acute lymphoblastic leukemia. Nat Genet. 2013;45: 186–190. doi:10.1038/ng.2508

4. Jones EW. Tackling the protease problem in Saccharomyces cerevisiae. Meth Enzymol. 1991;194: 428–453.

5. Eisinger DP, Dick FA, Trumpower BL. Qsr1p, a 60S ribosomal subunit protein, is required for joining of 40S and 60S subunits. Mol Cell Biol. American Society for Microbiology (ASM); 1997;17: 5136–5145.

6. Kallstrom G, Hedges J, Johnson A. The putative GTPases Nog1p and Lsg1p are required for 60S ribosomal subunit biogenesis and are localized to the nucleus and cytoplasm, respectively. Mol Cell Biol. American Society for Microbiology (ASM); 2003;23: 4344–4355. doi:10.1128/MCB.23.12.4344-4355.2003

7. Sengupta J, Bussiere C, Pallesen J, West M, Johnson AW, Frank J. Characterization of the nuclear export adaptor protein Nmd3 in association with the 60S ribosomal subunit. J Cell Biol. Rockefeller Univ Press; 2010;189: 1079–1086. doi:10.1083/jcb.201001124

8. Malyutin AG, Musalgaonkar S, Patchett S, Frank J, Johnson AW. Nmd3 is a structural mimic of eIF5A, and activates the cpGTPase Lsg1 during 60S ribosome biogenesis. EMBO J. 2017;: e201696012. doi:10.15252/embj.201696012
